# Intrinsic apoptosis and cytokine induction regulated in human tonsillar epithelial cells infected with enterovirus A71

**DOI:** 10.1371/journal.pone.0245529

**Published:** 2021-01-22

**Authors:** Menghuai Sun, Kunlong Yan, Chunyang Wang, Jiao Xing, Zhaojun Duan, Yu Jin, Carol J. Cardona, Zheng Xing

**Affiliations:** 1 Medical School and Jiangsu Provincial Key Laboratory of Medicine, Nanjing University, Nanjing, China; 2 Nanjing Children’s Hospital, Nanjing Medical University, Nanjing, China; 3 Clinical Medical College, Xi’an Medical University, Xi’an, China; 4 National Institute for Viral Disease Control and Prevention, Chinese Center for Disease Control and Prevention, Beijing, China; 5 Department of Veterinary Biomedical Sciences, College of Veterinary Medicine, University of Minnesota at Twin Cities, St. Paul, Minnesota, United States of America; University of Hong Kong, HONG KONG

## Abstract

Enterovirus A71 (EV-A71) has emerged as a clinically important neurotropic virus following poliovirus eradication. Recent studies have shown that human tonsillar epithelial cell lines (UT-SCC-60A and UT-SCC-60B) were susceptible to EV-A71, suggesting that human tonsillar crypt epithelium could be important in EV-A71 pathogenesis. However, the mechanism about how EV-A71 infects the upper oro-digestive tract remains largely unclear. In this study, we demonstrated that the human tonsillar epithelial cells infected with EV-A71 underwent apoptotic, in which cytochrome *c* was released from the mitochondria to the cytosol and caspase-9 was activated, while caspase-2 and -8 were not cleaved or activated during the infection. A selective inhibitor of caspase-9, Z-LEHD-FMK, inhibited the cleavage of the executioner caspase-3 and -7, indicating that only mitochondria-mediated intrinsic apoptotic pathway was activated in EV-A71-infected tonsillar epithelial cells. No evidence of pyroptosis or necroptosis was involved in the cell death. EV-A71 infection induced interferon, pro-inflammatory cytokines and chemokines, including IFN-β, IL-6, CCL5, and TNF-α in tonsillar epithelial cells, which may play a critical role in EV-A71-caused herpangina. Our data indicated that the induction of the cytokines was partially regulated by the mitogen-activated protein kinases (MAPKs) signaling pathway. The findings unveiled the host response to EV-A71 and its regulation mechanism, and will further our understanding the significance about the tonsillar crypt epithelium as the initial and primary portal in viral pathogenesis for EV-A71 infection.

## Introduction

Enterovirus A71 (EV-A71) is a single-stranded and positive-sense RNA virus from the *enterovirus* genus in the family *Picornaviridae* [1]. It is one of the major etiological pathogens of the hand, foot, and mouth diseases (HFMD), which is defined as a mild and self-limiting disease characterized by skin or mucosal vesicles or rashes in limbs and oral cavity. However, serious neurological diseases and complications manifesting as encephalitis, aseptic meningitis, or brainstem encephalitis could occur in severe and fatal cases [2,3]. Since the initial identification of EV-A71 in 1969, outbreaks with EV-A71 have occurred periodically throughout the world, especially in the Asia-Pacific region [4–6]. In China alone, nearly half a million HFMD cases were caused by EV-A71 infection in 2008 [7]. In fact nowadays EV-A71 has emerged as the most important enterovirus associated with neurological complications after a significant decrease and possible eradication of poliovirus worldwide [8].

The pathogenesis of human EV-A71 infection remains to be less understood. Presumably the virus enters the bloodstream in tissues or organs of the initial or primary site causing viremia, which may lead to neuroinvasion similar to the model with poliovirus. However, the entry site for EV-A71 into bloodstream remains controversial. The rate of viral isolation and detection from throat swabs appeared to be significantly higher than that from rectal swabs or feces, suggesting that the upper digestive tract may be a more important invasion target than the lower one [9,10]. Furthermore, a histopathologic study revealed that viral antigens and/or RNAs of EV-A71 were localized to squamous epithelium lining the tonsillar crypts but not in other parts of the gastrointestinal tract [11]. Recently, reports showed that human tonsillar epithelial cells supported EV-A71 replication and displayed innate antiviral immunity *in vitro* [12]. Clinically distinctive blebs in pharynx could be identified in patients with EV-A71 infection [13]. Thus we hypothesize that the human tonsillar crypt epithelium may be one of the major portals for EV-A71 initial or primary replication, playing significant roles in viral invasion into the circulatory and nervous system as well as shedding for viral spreading.

The paired palatine tonsils, located at the transition of the mouth to the oropharynx, the common entry of both the gastrointestinal and the respiratory tract, are the tissues exposed first to contact nasally and orally with various foreign microbes and substances in the air and in food [14]. As lymphoepithelial organism, palatine tonsils belong to the integrated mucosal immune system of the pharynx [15], which are analogous to organized lymphoid tissues in the gut [16] and the lung [17]. The tonsillar crypt epithelium is a modified form of the stratified squamous epithelium that covers the oropharynx including the outer surface of the tonsil [18]. The reticulated crypt epithelium, also called lymphoepithelium [19], plays a key role in the initiation of immune responses in the palatine tonsils [20].

Clinically, studies on palatine tonsil samples from elective tonsillectomy patients revealed that interferon-gamma (IFN-γ) and T-box expressed in T cells (T-bet) were upregulated following rhinovirus or parainfluenza virus infection [21]. Previous studies have also shown that Epstein–Barr virus (EBV) infection of tonsillar B cells led to a reduced cell surface expression of C-C Motif Chemokine Receptor 7 (CCR7) and C-X-C Motif Chemokine Receptor 5 (CXCR5), as well as to altered expression of several chemokine receptors and chemokines [22]. Additionally, tumor necrosis factor-alpha (TNF-α) and interleukin-1 alpha (IL-1α) production in tonsillar tissue cultures increased upon infection with human immunodeficiency virus-1 (HIV-1), which was found to have an impact on both innate and adaptive immune response [23]. Little is known, however, about how the tonsillar crypt epithelial cells respond to EV-A71 and how the response is regulated during EV-A71 infection.

Human tonsillar epithelial cells, which expressed SCARB2 and PSGL-1, viral receptors for EV-A71, were susceptible to EV-A71 as reported in a previous study [12]. IFN-α, IL8, IL-1β, IL-6 and IL-12p40 were induced and regulated by PI3K/AKT, p38, ERK1/2, and JNK1/2. In this report we showed that the human tonsillar epithelial cell lines UT-SCC-60A and UT-SCC-60B underwent apoptotic, which was exclusively intrinsic due to the process dependent on the release of cytochrome *c* into the cytosol upon EV-A71-infection. In response to EV-A71 infection, we analyzed a different set of antiviral or proinflammatory cytokines induced in the human tonsillar epithelial cells, including IFN-β andCCL5 and we showed that the response was partially regulated by MAPKs. Our data demonstrated that the tonsillar crypt epithelium may act as primary replicative tissues and play a critical role in EV-A71 viral pathogenesis in humans.

## Materials and methods

### Cells and virus

The human tonsillar epithelial cell lines, UT-SCC-60A and UTSCC-60B, were prepared at Dr. Reidar Grénman’s laboratory at Department of Otorhinolaryngology-Head and Neck Surgery, Turku University, Finland [24,25], which were isolated from a human tonsil with squamous cell carcinoma. The original tissues of the UT-SCC-60A and UT-SCC-60B cells came from the left tonsil and a lymph node in the neck, respectively. These cell lines were maintained in the Roswell Park Memorial Institute, cultured in RPMI 1640 medium (Gibco, Carlsbad, CA) supplemented with 10% fetal bovine serum (FBS) (Gibco). African green monkey kidney epithelial Vero cell line was purchased from the Cell Bank of Chinese Academy Sciences (Shanghai, China). The cells were maintained in Dulbecco’s modified Eagle’s medium (DMEM) with high glucose (Gibco) supplemented with 10% FBS. The cells were cultured at 37°C in a humidified atmosphere with 5% CO_2_. EV-A71 Fuyang-0805 strain (NCBI accession number: FJ439769), which belongs to the C4a cluster of the C4 subgenotype as verified by sequence analysis of the VP1 region.

### Virus preparation and titration

Monolayers of Vero cells at 80–90% confluence were infected with EV-A71 at an MOI of 5. After 2-hr adsorption, the cell monolayers were washed three times with phosphate buffer saline (PBS) and incubated in DMEM supplemented with 2% FBS at 37°C with 5% CO_2_. The virus stocks were prepared from supernatants of the Vero cells infected with the virus when significant cytopathogenic effects (CPE) appeared. The supernatants were clarified by centrifugation (12,000*g*), aliquoted, and stored at -80°C. Virus titers were determined in Vero cells by measuring the 50% tissue cell infectious dose (TCID_50_). For the TCID_50_ assay, serially diluted viruses from 10^−1^ to 10^−9^ in DMEM were inoculated to Vero cells cultured in 96-well plates and the cells were incubated for 7 days at 37°C. The TCID_50_ was calculated using the formula: Log_10_ TCID_50_ = *L*-*d*×(*s*-0.5), where *L* is the log of the lowest dilution, *d* is the difference between dilution steps, and *s* is the sum of the proportion of CPE-positive wells.

### Morphological observation and analysis

The UT-SCC-60A and UT-SCC-60B cells were infected with EV-A71 at an MOI of 5 and observed daily under a light microscopy for appearance of cytopathic effect (CPE). The images of the UT-SCC-60A and UT-SCC-60B cell monolayers at various time points after infection were captured by phase-contrast microscopy.

### Cell viability test

The cell viability was measured by a cell counting kit-8 (CCK-8, Beyotime Institute of Biotechnology, Shanghai, China). Briefly, the UT-SCC-60A and UT-SCC-60B cells were seeded into 96-well plates at a density of 2×10^3^ cells/well 16 hrs prior to infection. The cells were infected (MOI = 5) with EV-A71 and cultured for 72 hrs at 37°C. During this period, 10 μl of CCK-8 solution was added to each well at various time points after infection. After incubating CCK-8 with the cell cultures at 37°C for 30 mins in the incubator, the optical density (OD) of the supernatant in the culture was measured at 450 nm with a Multiskan FC (Thermo Fisher Scientific). The survival rate of cells was calculated and expressed as the ratio of the absorbance at OD_450_ (A_450_) of the infected cells to that of the uninfected cells. The assay was performed in triplicates for each time points.

### Quantitative real-time PCR

Total RNA was extracted from the UT-SCC-60A and UT-SCC-60B cells by TRIzol^TM^ Reagent (Invitrogen, Carlsbad, CA) following the manufacturer’s protocol. The quantity and purity of total RNA were evaluated by analyses with a Nanodrop 2000 spectrophotometer (Thermo Fisher Scientific). The cDNA was synthesized using SuperScript^TM^ IV VILO^TM^ Master Mix (Invitrogen). Real-time PCR was carried out using specific primers for each gene of interest. Reactions consisted of 10 μL 2×Power SYBR Green PCR Master Mix (ABI, Foster City, CA) in 20 μL and quantitative PCR was performed with QuantStudio™ 5 Real-Time PCR System (ABI). The 2^-ΔΔCt^ method was used to normalize and quantify relative fold change of gene copy numbers. Data were calculated as fold change (2^-ΔΔCt^), which was the copy number of corresponding gene transcripts normalized to an internal control, glyceraldehyde-3-phosphate dehydrogenase (GAPDH). For plotting EV-A71 replicative dynamics, the viral RNA fragments corresponding to nucleotides 2462–2635 of the EV-A71 Fuyang-0805 strain were adjusted to a concentration gradient (1×10^1^ to 1×10^8^ copies/μl) and used as a standard control to calculate the copy number of viral RNA. Quantitative PCR of the EV-A71 genes was also performed using the 2×Power SYBR Green PCR Master Mix (ABI). All primers used in this study were listed in S1 Table.

### Protein extraction and western blot analysis

Infected and uninfected cells were lysed at indicated time points using the Radio Immunoprecipitation Assay (RIPA) lysis buffer (Beyotime) supplemented with protease and phosphatase inhibitors (Roche, Basel, Switzerland) to obtain the whole cell lysates. For the cytosol and mitochondrial fractions, we performed preparations using a Mitochondria Isolation Kit for Mammalian Cells (Thermo Fisher Scientific). Proteins of different fractions were denatured and separated using sodium dodecyl sulfate-polyacrylamide gel electrophoresis (SDS-PAGE) prior to their immobilization by transfer to a polyvinylidene difluoride (PVDF) membrane (Bio-Rad, Hercules, CA). After blocking with 5% nonfat milk in Tris-buffered saline and Tween-20 (TBST), the membrane was incubated with diluted primary antibodies overnight at 4°C. All primary antibodies used in western blot analyses were purchased from Cell Signaling Technology (Boston, MA). After washing three times with TBST, the membrane was incubated with diluted horseradish peroxidase (HRP)-conjugated secondary antibodies. Signals on the blots were developed using Immuno-Star™ HRP Substrate and images were captured using a ChemiDoc™ XRS+ imaging system (Bio-Rad).

### Caspase activity analysis

Cytosolic lysates of the infected and uninfected cells were prepared as described above and normalized by a Bradford assay with reagents from Beyotime. Caspase-3, -8, and -9 activities were detected by using corresponding caspase activity assay kits (Beyotime) and performed according to the kit instructions. Ac-DEVD-pNA, Ac-IETD-pNA, and Ac-LEHD-pNA were used as the substrates for caspase-3, -8 and -9, respectively. Absorbance at OD_405_ (A_405nm_) was determined in a Multiskan FC (Thermo Fisher Scientific) and the data were expressed as fold change relative to the control.

### Enzyme linked immune sorbent assay (ELISA) for cytokines

Cell culture supernatants were harvested at indicated time points from the infected and uninfected UT-SCC-60A and UT-SCC-60B cells, pre-treated with or without inhibitors, by centrifugation at 1,000 *g* for 15 min at 4°C. Quantitative measurements of the cytokines were performed in 96-well plates using solid-phase sandwich-type ELISA kits (RayBiotech, Norcross, GA) according to the manufacturer’s instructions. The absorbance was read at 450 nm using the Multiskan FC. Serial dilutions of the standard controls were prepared and used to plot a standard curve of absorbance at 450 nm utilizing linear regression analysis. The assay was repeated three times for each sample.

### Immunofluorescence analysis

Infected or uninfected cells were fixed with 4% paraformaldehyde at room temperature for 30 min and permeabilized with 0.1% Triton X-100 on ice for another 10 min, followed by three washes with 1×PBS, then blocked with 5% BSA at 37°C for 1 hr. The cells were incubated with primary antibodies diluted in PBS/Tween 20 (PBST) containing 1% BSA at 4°C overnight. The primary antibodies included mouse anti-EV-A71 structural protein VP1 and mouse anti-cytokeratin 8+18 antibodies that were purchased from Abcam (Cambridge, MA). After washes with PBST three times, the cells were incubated with anti-mouse IgG conjugated with either Alexa Fluor 488^TM^ or Alexa Fluor 568^TM^ (Molecular Probes, Eugene, OR) at a 1:500 dilution for another 1 hr at 37°C. The cells were then washed and incubated with a solution of DAPI at 5 μg/ml diluted in PBS for 10 min. After three washes in PBST, the cells were covered with one droplet of Prolong^TM^ Gold Antifade Mountant (Molecular Probes) and observed under a FV3000 Confocal Laser Scanning Microscope (Olympus, Tokyo, Japan).

### TUNEL assay

TUNEL assays were performed on the UT-SCC-60A and UT-SCC-60B cells using an *In Situ* Cell Death Detection Kit with Fluorescein (Roche, Basel, Switzerland) according to the manufacturer’s instructions. The fixed cells were treated with DNase I as positive controls. Samples were incubated with the TUNEL reaction mixture for 1 hr at 37°C in the dark and then washed twice in PBS. The condensed or fragmented nuclei of apoptotic cells were observed under an FV3000 Confocal Laser Scanning Microscope.

### Inhibitors for the ERK1/2, JNK, and p38 kinases

U0126, SP600125, SB203580, inhibitors of the ERK1/2, JNK, and p38, respectively, were all purchased from Beyotime. The three chemicals were dissolved in DMSO at stock concentrations of 10, 15, and 10 mg/mL, respectively, and stored at -30°C before use.

### Statistical analysis

The two-tailed Student’s *t*-test was used to analyze the data. The data shown were the means ± standard deviations (SD) from three independent experiments. p values of <0.05 were considered statistically significant.

## Results

### Cell death caused by infection of EV-A71 in human tonsillar epithelial cells

**Fig 1:**
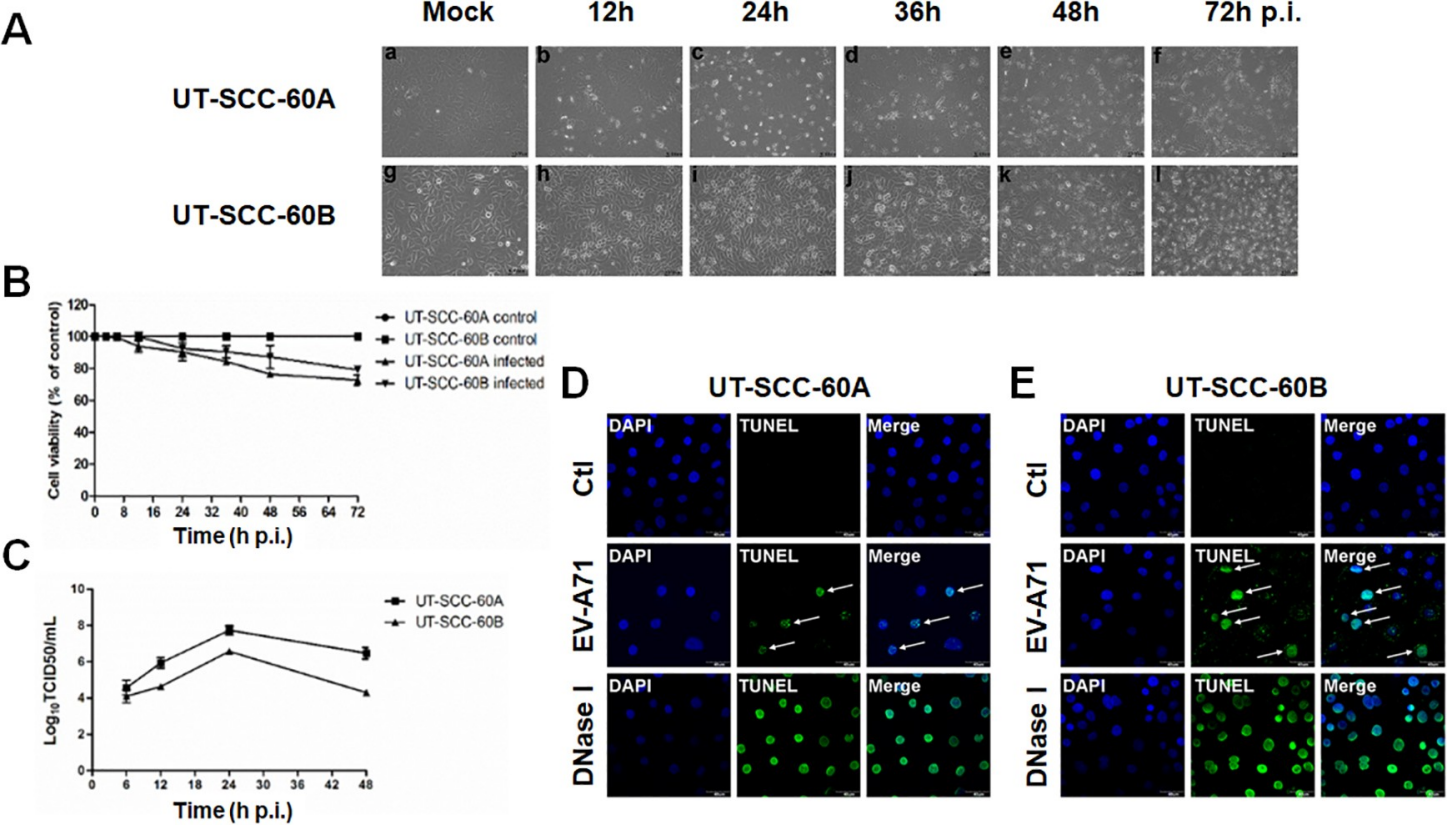
Cell death induced in human tonsillar epithelial cells infected with EV-A71. (**A**) Cytopathic Effects of EV-A71-infected UT-SCC-60A and UT-SCC-60B cells. Human tonsillar epithelial cell lines, UT-SCC-60A and UT-SCC-60B, were infected with EV-A71 at an MOI of 5 and morphology of the cells was subjected to observation under light microscopy starting 24 hrs after infection. Magnification, ×100. (**B**) Cell viabilities of the infected UT-SCC-60A and UT-SCC-60B cells were assessed by the CCK-8 assay as described in Methods. (**C**) Titration of infectious viral titers in infected UT-SCC-60A and UT-SCC-60B cells. Supernatants from infected cells were collected at various time points p.i. Infectious viral titers were measured by a standard TCID_50_ assay. (**D** & **E**) Chromatin DNA breakage was detected in infected UT-SCC-60A and UT-SCC-60B cells. Uninfected and infected cells were on glass slides, fixed at 48 h p.i. and subjected to a TUNEL assay. The green fluorescence indicates DNA strand breaks generated in cell death. Images were captured using an Olympus confocal laser scanning microscope. Ctl, uninfected cells. Magnification, ×600.

To study how human tonsillar epithelial cells respond to EV-A71 infection, we inoculated UT-SCC-60A and UT-SCC-60B cells with EV-A71 at a multiplicity of infection (MOI) of 5. The CPE occurring in cells was monitored microscopically at 12, 24, 36, 48, and 72 hrs post-infection (p.i.). As shown in Fig 1A, the infected UT-SCC-60A and UT-SCC-60B cells underwent apparent morphological changes, including cell rounding, swelling, and detachment from the culture monolayer, and eventually burst into granules with the whole cell monolayers devastated after 48 and 72 hrs p.i., respectively. The cell death was confirmed and quantitated by examining cell viabilities with or without viral infection, which were assessed by a cell counting kit-8 (CCK-8) assay. The cell viability in the infected cells decreased up to 70% (UT-SCC-60A) or 80% (UT-SCC-60B) at 72 hrs p.i. in comparison with the uninfected cells (Fig 1B).

The cell death was caused apparently by EV-A71 infection, which was demonstrated by EV-A71 replicative kinetics in the human tonsillar epithelial cells. The titers of the infectious virus in the cell cultures were titrated and the EV-A71 yield increased throughout the infection when it peaked at 24 hrs p.i. up to 7.7 and 6.5 Log_10_ TCID_50_/mL in UT-SCC-60A and UT-SCC-60B cells, respectively (Fig 1C). The viral yield decreased thereafter, probably due to extensive cell death. TUNEL assay was performed to detect DNA strand breaks during EV-A71 infection. The results showed positive TUNEL staining observed at 48 hrs p.i. in the two cell lines infected with EV-A71 (Fig 1D and 1E). Collectively, our data demonstrate that human tonsillar epithelial cells underwent extensive cell death during a productive EV-A71 infection of in the two human tonsillar epithelial cell lines and the cell death could be apoptotic.

### EV-A71 induced apoptosis in human tonsillar epithelial cells

**Fig 2:**
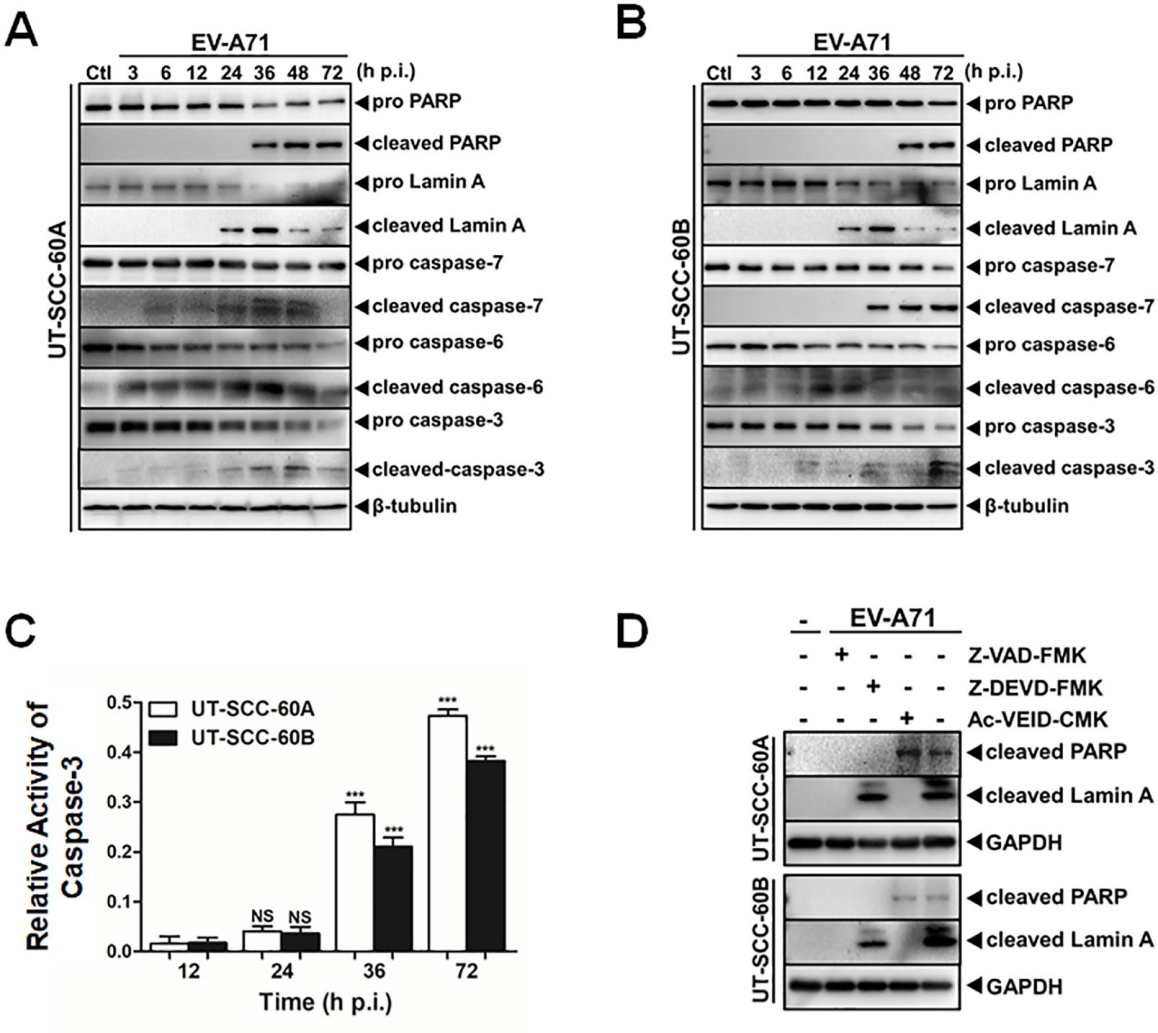
Induction of apoptosis in EV-A71-infected human tonsillar epithelial cells. (**A** & **B**) Cleavage of executioner pro-caspases and substrates PARP and Lamin A. Cell lysates were prepared from EV-A71-infected and uninfected UT-SCC-60A (**A**) and UT-SCC-60B (**B**) cells at indicated time points. Western blot analyses were performed for detection of the caspases as well as substrates with specific antibodies. β-tublin was used as an internal control. (**C**) Relative caspase-3 activities in UT-SCC-60A and UT-SCC-60B cells infected with EV-A71 detected at indicated time points. Ac-DEVD-pNA was used as the substrate for caspase-3. The data were expressed as mean fold of values at A_405_ relative to uninfected controls. The assay for each sample was repeated twice (*t*-test; *p < 0.05, **p < 0.01, ***p < 0.001). (**D**) Effect of caspase inhibitors on EV-A71-induced cleavage of PARP and Lamin A. UT-SCC-60A and UT-SCC-60B cells were pre-treated with a pan-caspase inhibitor (Z-VAD-FMK), caspase-3-specific inhibitor (Z-DEVD-FMK), or caspase-6-specific inhibitor (Ac-VEID-CMK) for 12 hrs, followed by EV-A71 infection for another 72 hrs. Cell lysates were prepared for western blot analyses with specific antibodies to detect cleavage of the caspases and substrates.

We showed chromatin DNA breakage detected in infected UT-SCC-60A and UT-SCC-60B cells by TUNEL assay. To further confirm apoptosis was triggered after infection, we measured caspase activation and substrate cleavage by western blot analyses and detected that the cleavage of poly (ADP-ribose) polymerase (PARP), one of the main cleavage targets of caspase-3 [26], which increased during the late stage of infection in the two cell lines (Fig 2A and 2B). In addition, Lamin A, a marker for caspase-6 activation, was cleaved as well after EV-A71 infection, which was apparently associated to nuclear dysregulation and subsequent cell death [27] (Fig 2A and 2B). The cleaved forms of executioner caspase-3, -6, and -7 were detected in both cell lines simultaneously (Fig 2A and 2B), confirming that apoptosis was induced in human tonsillar epithelial cells after EV-A71 infection. We further examined caspase-3 activity in the infected cells by using a caspase-3 activity assay kit and found that it increased in a time-dependent manner in EV-A71-infected cells (Fig 2C).

To verify the specific caspases and substrate involved in EV-A71 infection, we pre-treated the cells with a pan-caspase inhibitor (Z-VAD-FMK, 50 μM), a selective caspase-3 inhibitor (Z-DEVD-FMK, 100 μM), and a specific caspase-6 inhibitor (Ac-VEID-CMK, 100 μM), respectively, followed by EV-A71 infection. After a 72-hr inoculation, the whole cell lysates were collected and analyzed by western blot analyses. The results showed that the inhibitor Z-VAD-FMK completely blocked EV-A71-induced cleavage of PARP and Lamin A in two types of the cells (Fig 2D). However, when treated with Z-DEVD-FMK specific for caspase-3, no significant change of the cleaved Lamin A protein was observed compared with the untreated EV-A71-infected cells, indicating that Z-DEVD-FMK hindered EV-A71-induced cleavage of PARP but had no effect on the cleavage of Lamin A in both cell types. In contrast, Ac-VEID-CMK inhibited cleavage of Lamin A but had no impact on cleavage of PARP (Fig 2D). These data showed the specificity of the substrates to the executioner caspases and, while PARP is susceptible to caspase-3, Lamin A is cleaved by caspase-6 in human tonsillar epithelial cells. Both caspases were activated upon EV-A71 infection.

### Intrinsic but not extrinsic pathway involved in EV-A71-induced apoptosis

**Fig 3:**
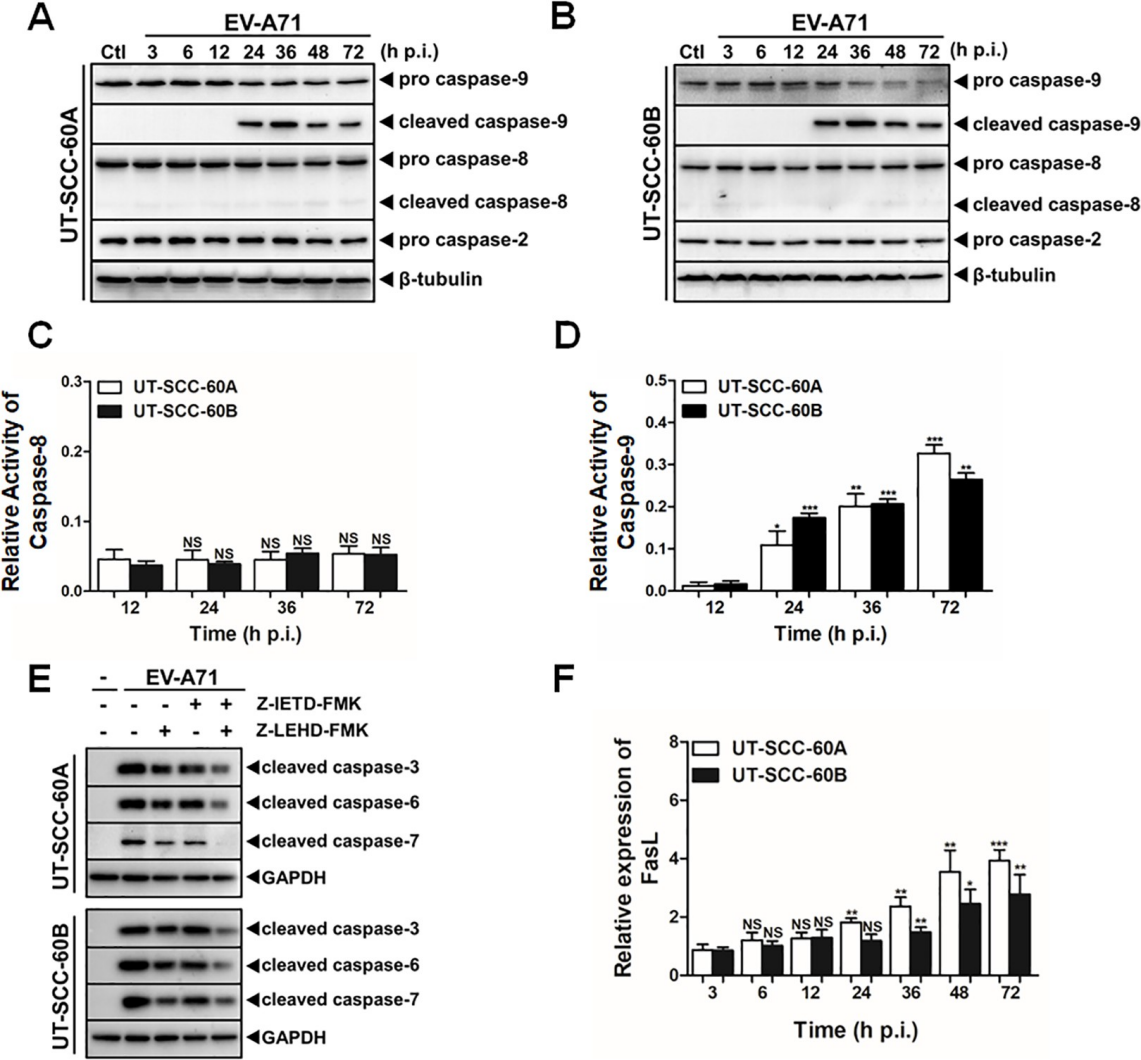
Intrinsic apoptosis induced in EV-A71-infected human tonsillar epithelial cells. (**A** & **B**) Activation of caspase-9, but not caspase-8 or -2, in infected human tonsillar epithelial cells. UT-SCC-60A (**A**) and UT-SCC-60B (**B**) cells were infected with EV-A71 at an MOI of 5 and cell lysates were prepared at indicated time points for western blot analyses with specific antibodies. Ctl, control. (**C** & **D**) Enhancement of caspase-9 activity, but not caspase-8 activity, induced by EV-A71 infection. The cells were pre-treated with the substrate chemicals, IETD-pNA and LEHD-pNA for caspase-8 and -9, respectively, and the lysates were prepared for measurements from 12 to 72 hrs p.i. The data were expressed as mean relative fold of the A_405_ values. The assay for each sample was repeated in triplicates. (**E**) Inhibition of caspase-9 activation blocked the cleavage of caspase-3, -6, and -7 effectively in EV-A71-infected UT-SCC-60A and UT-SCC-60B cells. Both cell lines were pre-treated with a caspase-8 inhibitor (Z-LETD-FMK, 50 μmol/L) or a caspase-9 inhibitor (Z-LEHD-FMK, 50 μmol/L), followed by EV-A71 infection for another 48 hrs. Cell lysates were prepared and analyzed by western blot analyses with antibodies for cleaved caspase-3, -6, and -7. (**F**) No significant induction of FasL in infected UT-SCC-60A and UT-SCC-60B cells. Total RNA was prepared from infected UT-SCC-60A and UT-SCC-60B cells at various time points for real-time PCR with primers for FasL to measure its fold change in response to infection. Values represent mean ± SD from triplicates of independent experiments (*t*-test; NS, no significance; *p<0.05, **p<0.01, ***p<0.001).

To identify upstream initiator caspases, we examined whether pro-caspase-8 or -9 was activated and involved in induced apoptosis. As shown in Fig 3A and 3B, cleaved/activated caspase-9 increased after EV-A71 infection from 24 to 72 hrs p.i. in both cell lines. However, the levels of pro-caspase-8 were unchanged and the cleaved form of caspase-8 was never detected throughout the infection, suggesting that the infection only induced the activation of a caspase-9-dependent intrinsic apoptotic pathway. This differential pattern of caspase activation was in consistency with our previous observation in human astrocytes infected with EV-A71. Likely cell types dictate the pathway of apoptosis to be activated. In HT-29 cells, however, cleavage of both caspase-8 and -9 was exhibited in response to EV-A71 [28].

We also used the caspase activity test kit to measure activities of both caspase-8 and -9 for confirmation in the two lines of the cells. We found that the activity of caspase-9 increased starting from 24 hrs p.i. but no significant change was observed in the activity of caspase-8 in both cell types (Fig 3C and 3D).

To further confirm that only caspase-9 was activated, the cells were pre-treated with Z-LETD-FMK and Z-LEHD-FMK, selective inhibitor of caspase-8 and -9, respectively, followed by EV-A71 infection. The results showed that Z-LEHD-FMK effectively suppressed the cleavage of pro-caspase-3, -6 and -7, while Z-LETD-FMK only mildly hampered the activation of these caspases (Fig 3E). The inhibitory effect of Z-LETD-FMK might be attributed as some nonspecific effects on other caspases. Taken together, these results suggest that caspase-9 may be the upstream initiator caspase that was cleaved and activated, which subsequently activated pro-caspase-3, -6, and -7 in the human tonsillar epithelial cell lines infected with EV-A71.

Caspase-2 is also a well-established initiator caspase for the intrinsic apoptotic pathway [29,30]. From our observation as shown in Fig 3A and 3B, the level of pro-caspase-2 was not changed and cleaved caspase-2 was not observed, indicating that caspase-2 was not involved in the cell death. Our data showed that caspase-9 activation was likely the only initiator activated in the apoptotic pathway in EV-A71-infected tonsillar epithelial cells.

### EV-A71 induced cytochrome *c* release and regulated expression of Bcl-2 and IAP family members

**Fig 4:**
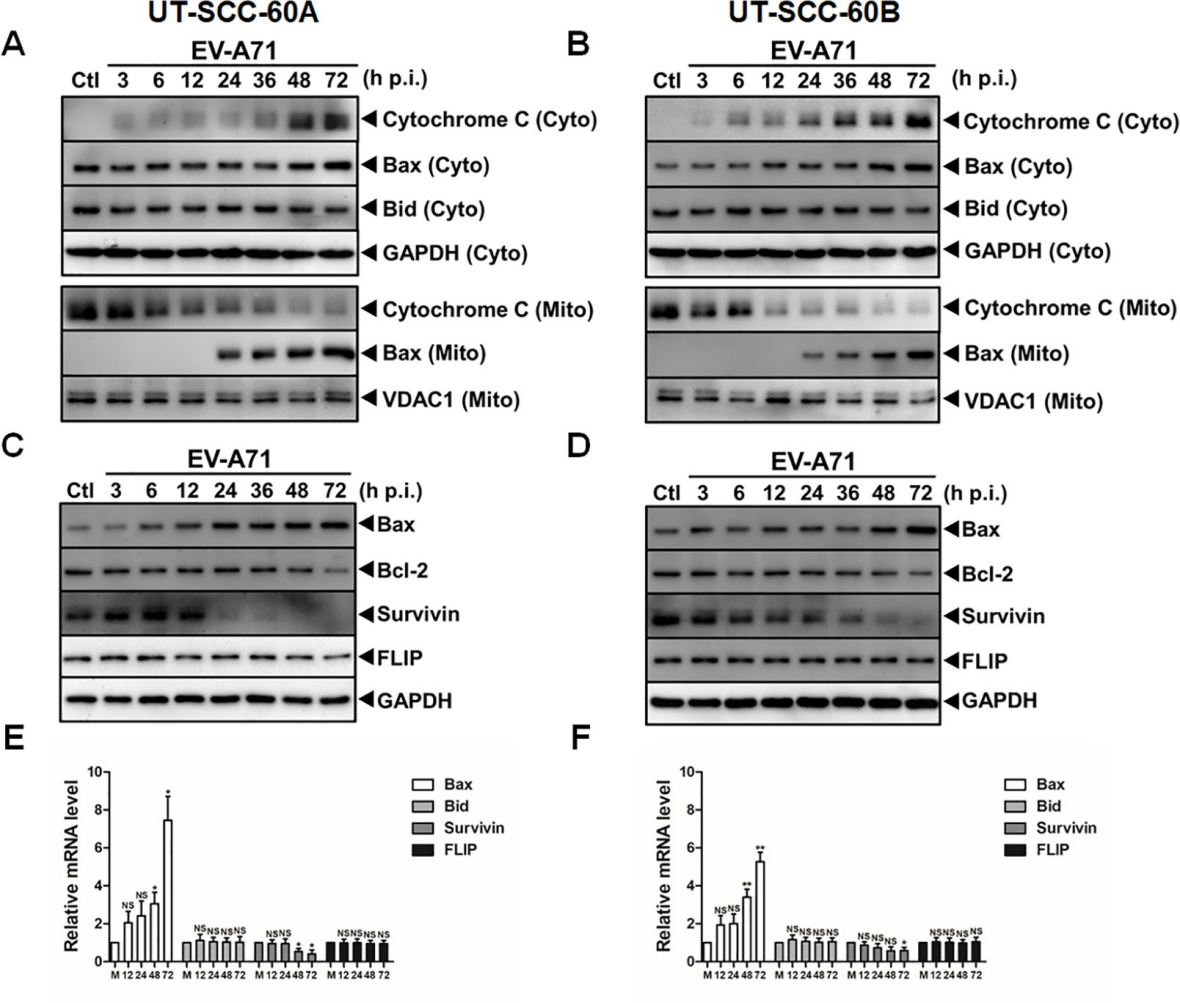
Release of cytochrome c and upregulation of Bax in EV-A71-infected human tonsillar epithelial cells. (**A** & **B**) Upregulation of Bax and release of cytochrome *c*. Cytosolic (Cyto) and mitochondrial (Mito) fractions of the cell lysates were prepared from uninfected (Ctl) and infected cells at various time points p.i. The levels of Bax and cytochrome *c* were determined by western blot analyses. GAPDH and VDAC1 were detected as internal control for proteins in the cytosolic and mitochondrial fractions, respectively. The assays were repeated at least twice. (**C** & **D**) Down-regulation of Bcl-2 and survivin and upregulation of Bax in EV-A71-infected UT-SCC-60A and UT-SCC-60B cells. Uninfected (Ctl) and infected cells were infected with EV-A71 and total cell lysates were collected and analyzed by western blot analyses with specific antibodies. (**E & F**) Regulation of the apoptotic genes in UT-SCC-60A (**E**) and UT-SCC-60B (**F**) cell lines infected with EV-A71. Total RNA was prepared at the indicated time points p.i. for quantitative RT-PCR using primers specific for Bax, Bid, Survivin or FLIP. Values represent mean ± SD from triplicates of independent experiments (*t*-test; NS, no significance; *p<0.05, **p<0.01, ***p<0.001).

We sought to identify the mechanism by which the intrinsic apoptosis was induced and detected that cytochrome *c* was released from the mitochondrial membrane into the cytosol in the cells infected with EV-A71. As shown in Fig 4A and 4B, decreased levels of cytochrome *c* were observed in the mitochondrial fraction, whilst the levels of cytochrome c increased in the cytosol at 12 and 24 hrs p.i. in the two cell lines, respectively. Along with these changes, the expression of Bax was upregulated in both the cytosolic and mitochondrial fractions after the infection (Fig 4A and 4B). It is likely that Bax was induced, which was further translocated from the cytosol to the mitochondrial membrane upon EV-A71 infection. The induction of Bax appeared to be intensive that we could see the levels of Bax upregulated in the whole cell lysates at the late stage of infection (Fig 4C and 4D).

Cytochrome *c* release can be promoted by tBid, a cleaved product of Bid and another pro-apoptotic Bcl-2 family member, onto the mitochondrial membrane. Our data showed that tBid, an active form of Bid, was present at a low level constitutively in the mitochondria of the tonsillar epithelial cells without infection. The levels of tBid remained unchanged throughout the infection, suggesting that the cytochrome *c* release was independent of tBid (data not shown). We did not observe a significant induction of Fas ligand (FasL), which could lead to no stimulation of Fas or activation of pro-caspase-8 (Fig 3F).

Activation of caspase signaling and apoptosis may also be promoted by downregulation of anti-apoptotic factors. We examined total cell lysates collected at various time points after infection and found that anti-apoptotic Bcl-2 was downregulated at 72 hrs p.i. and survivin declined at 24 and 72 hrs p.i. in both cell line (Fig 4C and 4D) while FLIP, an inhibitor of pro-caspase-8, remained unchanged. These data were further confirmed by measuring mRNA transcripts of these genes with RT-PCR (Fig 4E and 4F). Reduced Bcl-2 may promote cytochrome *c* release from the mitochondria. Executioner caspase-3 and -7 would be more active with a reduced level of survivin in the cytosol at the later stage of infection.

### Neither pyroptosis nor necroptosis was involved in the cell death of human tonsillar epithelial cells infected with EV-A71

**Fig 5:**
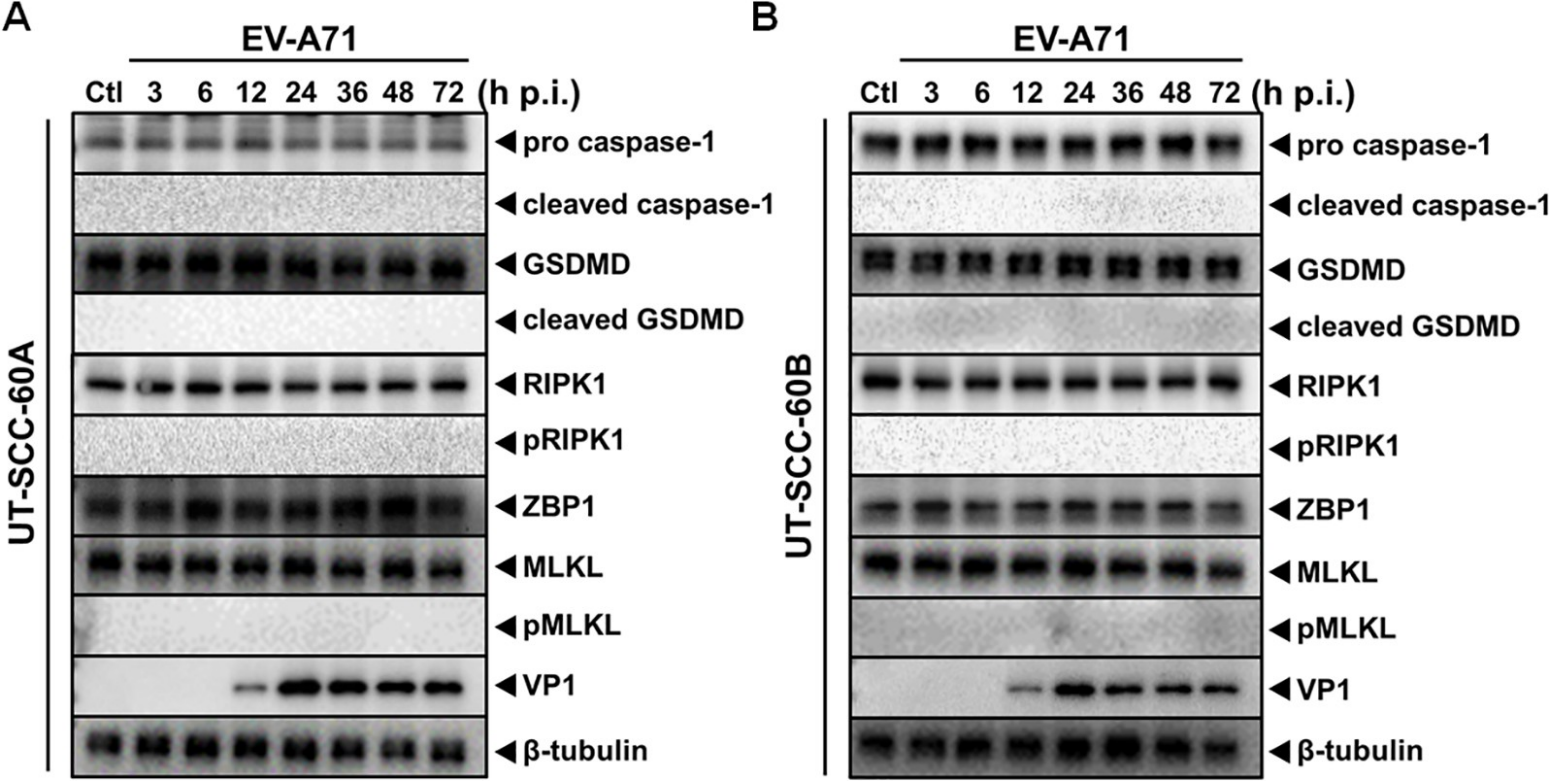
Pyroptosis and necroptosis were not induced in EV-A71-infected human tonsillar epithelial cells. (**A** & **B**) UT-SCC-60A and UT-SCC-60B cells were infected with EV-A71 at an MOI of 5 and cell lysates were prepared at indicated time points p.i. for western blot analysis with antibodies specific for proteins involved in pyroptosis (pro/cleaved caspase-1 and pro/cleaved GSDMD) or necroptosis (RIPK1/p-RIPK1, MLKL/p-MLKL, and ZBP1).

We tried to understand whether other mechanism may be involved in the cell death caused by EV-A71 in the human tonsillar epithelial cells. Cell lysates were analyzed from the cell cultures of both UT-SCC-60A and UT-SCC-60B lines at various time points after infection. No cleaved caspase-1 or cleaved gasdemin D (GSDMD) was detected indicating that pyroptosis was not induced. Necroptosis appeared not to have occurred since RIPK and MLKL were not phosphorylated or activated either in both cell lines (Fig 5A and 5B).

### Induction of cytokines in EV-A71-infected human tonsillar epithelial cells

**Fig 6:**
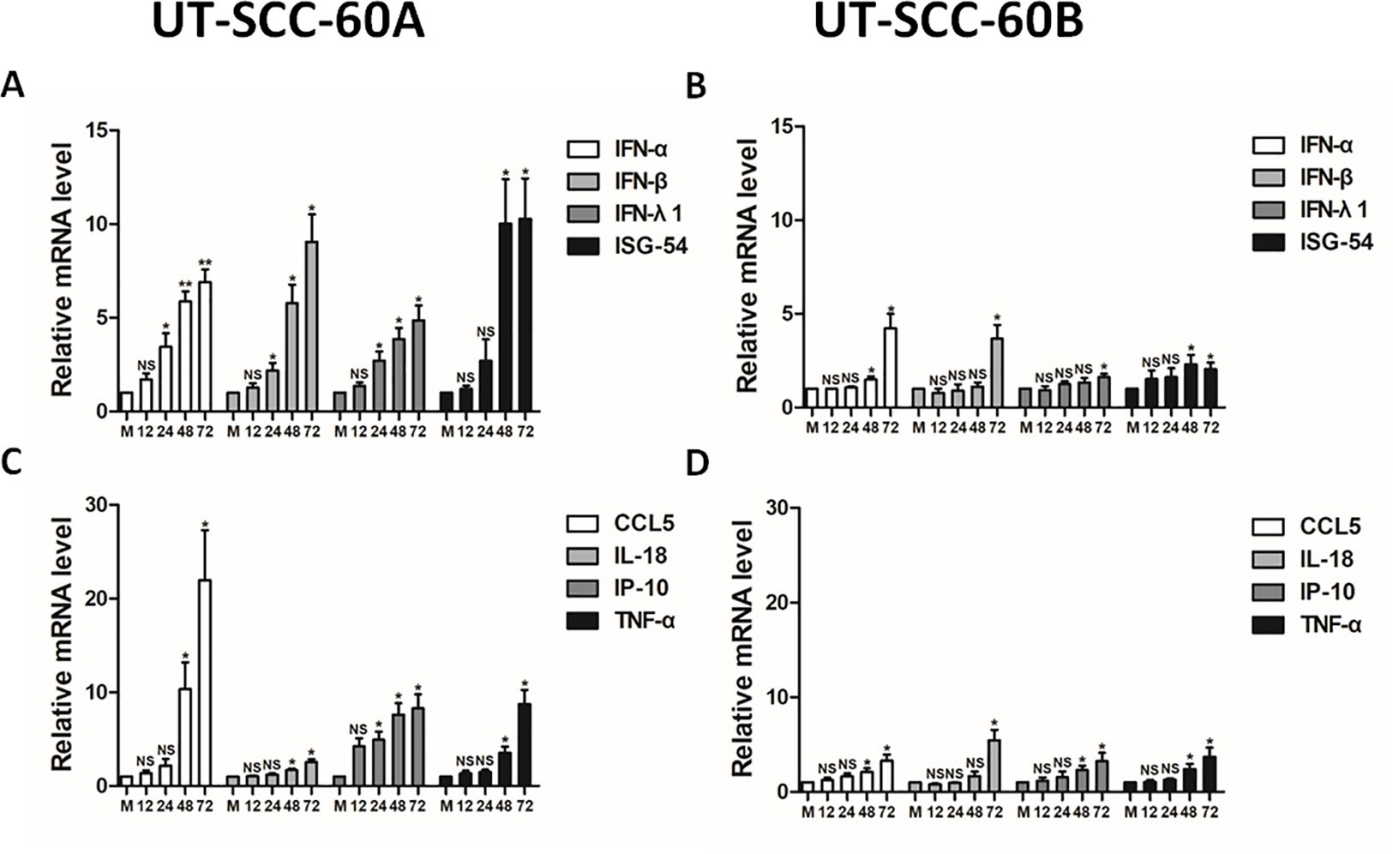
Induction of cytokine and chemokine gene transcription in EV-A71-infected tonsillar epithelial cells. UT-SCC-60A (**A** & **C**) and UT-SCC-60B (**B** & **D**) cells were infected with EV-A71 at an MOI of 5 and total RNA was prepared at the indicated time points for quantitative RT-PCR using primers specific for IFN-α, IFN-λ1, IFN-λ3, IL-1β, IL-8, IL-18, ISG-54, ISG-56, or IP-10. Values represent mean ± SD from triplicates of independent experiments (*t*-test; *p < 0.05, **p < 0.01).

To elucidate other host responses in EV-A71-infected tonsillar epithelial cells, we examined induction of interferon, proinflammatory cytokines, and chemokines in UT-SCC-60A and UT-SCC-60B cells. The results showed that the levels of IFN-α, IFN-β, IFN-λ1 and ISG-54transcripts increased in UT-SCC-60A cells, while these genes remained unchanged or changed marginally in UT-SCC-60B cells (Fig 6A and 6B). As for pro-inflammatory cytokines and chemokines, we observed that the transcripts of CCL5 (RANTES) increased significantly after infection whereas moderate increases of IL-18, IP-10 and TNF-α transcripts were detected in UT-SCC-60A cells. In contrast, all these inflammatory genes including CCL5 changed marginally in UT-SCC-60B cells (Fig 6C and 6D).

**Fig 7:**
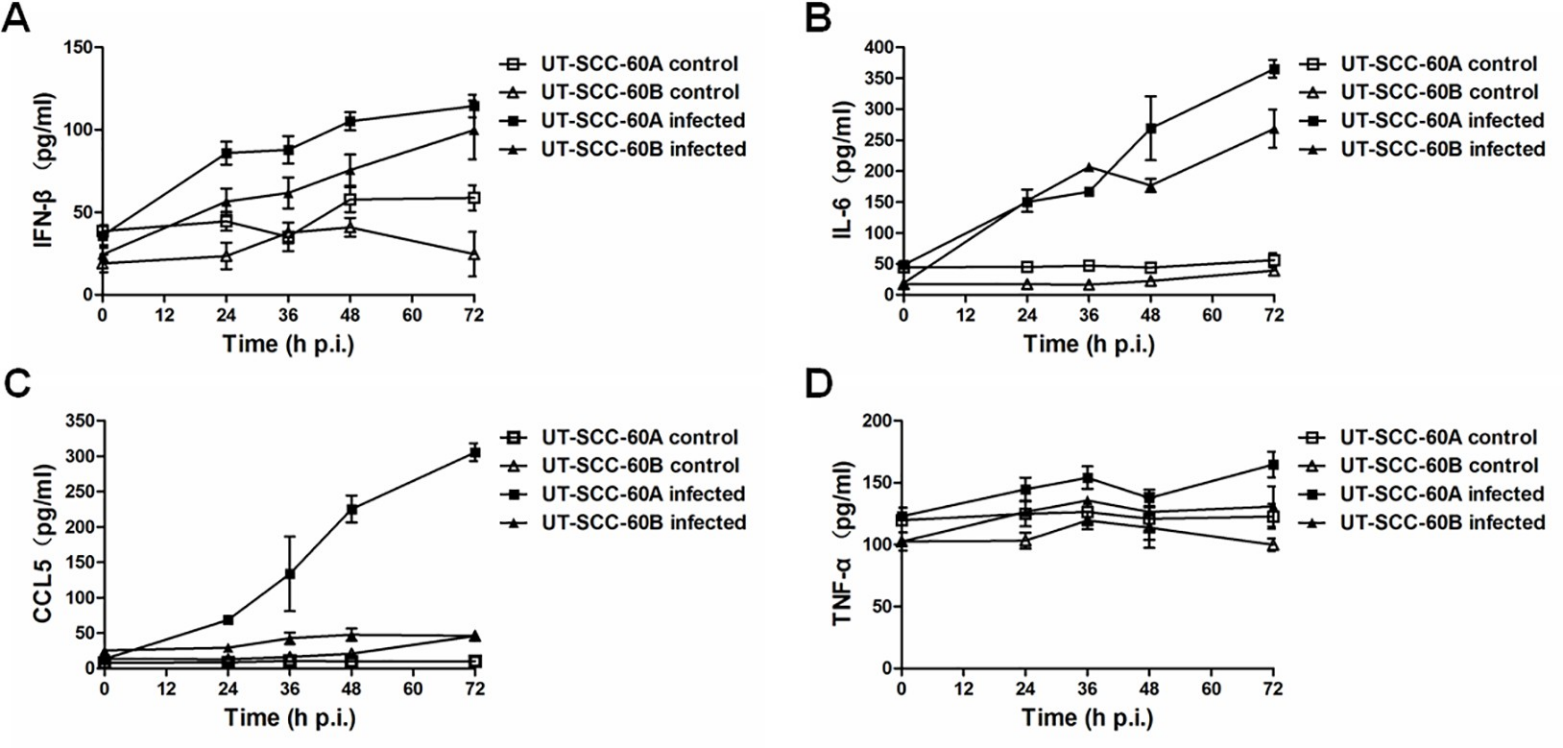
Induction of IFN-β and CCL5 in EV-A71-infected human tonsillar epithelial cells. UT-SCC-60A and UT-SCC-60B cells were infected with EV-A71 and total RNA was prepared for quantitative RT-PCR using primers specific for IFN-β (**A**), IL-6 (**C**), CCL5 (**E**), or TNF-α (**G**). The cultural media of both cell lines infected with EV-A71 were collected at various time points after infection for ELISA to measure the protein levels of IFN-β (**B**), IL-6 (**D**), CCL5 (**F**), and TNF-α (**H**). Values represent mean ± SD from triplicates of independent experiments (*t*-test; *p< 0.05, **p < 0.01).

To confirm the induction of these cytokines, we also measured the protein levels of IFN-β, IL-6, CCL5 and TNF-α from the culture medium with ELISA. As shown in Fig 7, elevation of IFN-β, IL-6, and CCL5 was detected in both UT-SCC-60A and UT-SCC-60B cells infected with EV-A71. Higher levels of IFN-β, IL-6, and CCL5 were detected in UT-SCC-60A than in UT-SCC-60B cells (Fig 7A–7C). The induction of TNF-α was not evident in both lines of the cells, consistent with its mild change as mRNA transcripts depicted in Fig 6. Both IL-1β and IL-18 were not detected in the cultural medium probably because no pyroptosis was induced in these cells. These results suggest that a robust interferon, proinflammatory cytokine, and chemokine induction occurred in human tonsillar epithelial cells infected with EV-A71. UT-SCC-60A cells were more responsive to EV-A71 infection than UT-SCC-60B cells. The difference in cytokine responses between the two cell lines is probably due to their distinctive origins.

### Effects of MAPKs on the induction of Cytokines in EV-A71-infected human tonsillar epithelial cells

**Fig 8:**
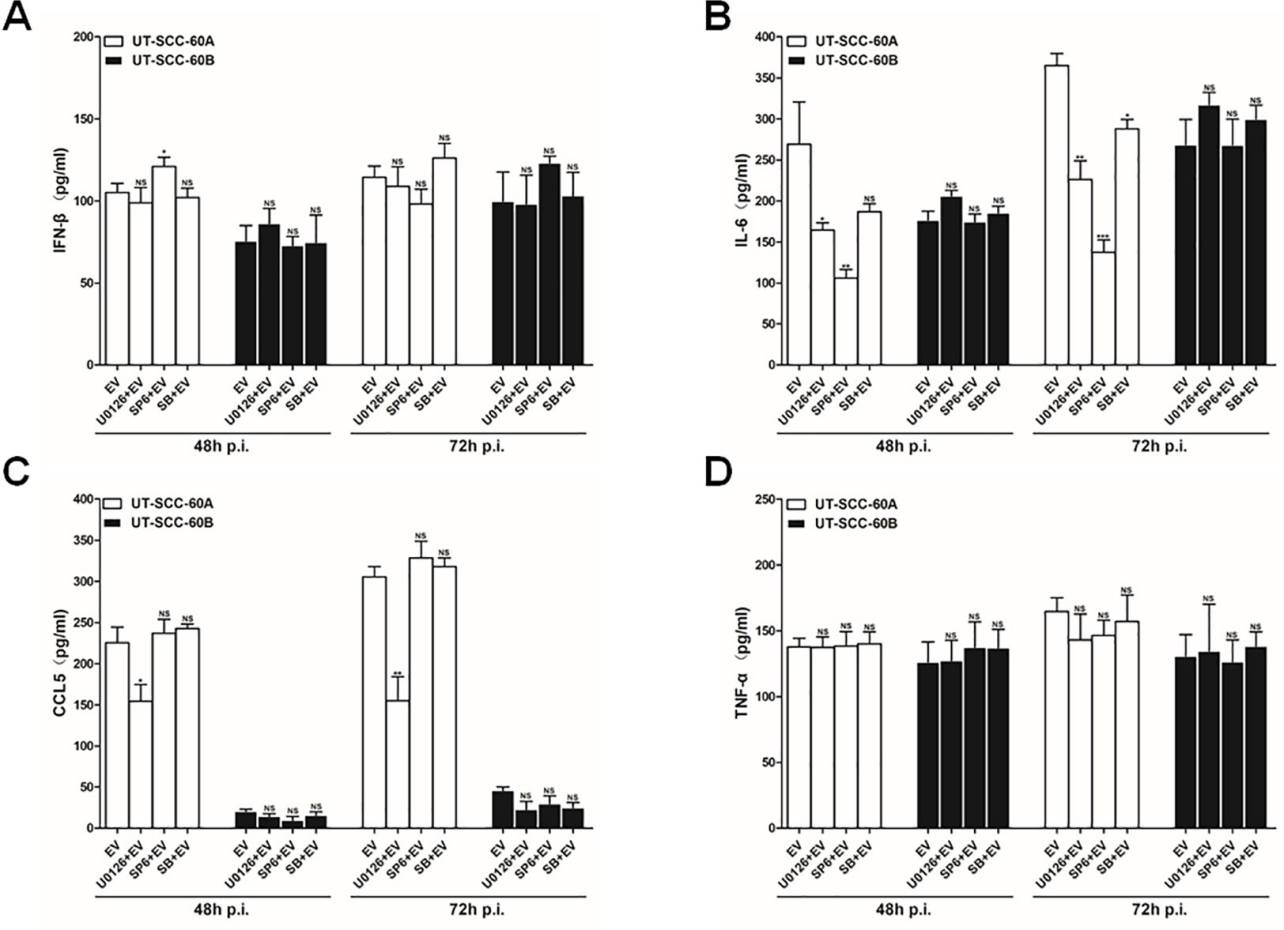
Regulation of cytokine induction by MAPKs in EV-A71-infected human tonsillar epithelial cells. UT-SCC-60A and UT-SCC-60B cells were pre-treated with inhibitors for ERK1/2 (U0126), JNK1/2 (SP600125), or p38 (SB203580) at 1 hr prior to EV-A71 infection. The cultural media were collected at 48 and 72 hrs p.i. for ELISA to measure the levels of IFN-β (**A**), IL-6 (**B**), CCL5 (**C**), and TNF-α (**D**). The experiments for each sample were repeated at least three times (*t*-test; NS, no significance. *p < 0.05, **p < 0.01).

We focused on a selected group of the antiviral and proinflammatory cytokines and tried to evaluate the role of MAPKs in their induction in EV-A71-infected human tonsillar epithelial cells. Culture supernatants were prepared from infected or uninfected UT-SCC-60A cells and UT-SCC-60B cells pre-treated with inhibitors specific for the extracellular signal-regulated kinases (ERKs) 1/2 (U0126), c-Jun N-terminal kinases (JNKs) 1/2 (SP600125), and p38 (SB203580), respectively. The culture supernatants were measured for induction of IFN-β, IL-6, CCL5, and TNF-α with ELISA kits. As shown in Fig 8A, although higher IFN-β was induced in UT-SCC-60A cells than in UT-SCC-60B cells, pre-treatment with U0126, SP600125, or SB203580 appeared not to impact the induction of IFN-β significantly, indicating that IFN-β was likely regulated by the signaling pathways other than MAPKs in EV-A71-infected tonsillar epithelial cells. On the other hand, in the presence of ERK1/2, JNK, and p38 inhibitors, especially the JNK inhibitor, the induction of IL-6 was evidently suppressed at 48 and 72 hrs p.i. in UT-SCC-60A cells (Fig 8B), indicating that the induction of IL-6 was dependent on ERK1/2, JNK, and p38 signaling in UT-SCC-60A cells. However, the levels of IL-6 in EV-A71-infected UT-SCC-60B cells remained unchanged or upregulated in the presence of the ERK1/2, JNK, and p38 inhibitors (Fig 6B), suggesting that IL-6 was refractory to regulation by MAPKs in UT-SCC-60B cells.

We observed that the levels of CCL5 decreased in the presence of U0126 at 48 and 72 hrs p.i. in both UT-SCC-60A and UT-SCC-60B cells, while SP600125 and SB203580 exerted little influence on the induction of CCL5, indicating that CCL5 induction was differentially regulated only by ERK1/2 signaling in the tonsillar epithelial cells (Fig 8C). In sum, we concluded that ERK1/2, JNK, and p38 signaling of MAPKs played a significant role in the differential induction of the selected group of antiviral or proinflammatory cytokines, which could be critical to viral pathogenicity in EV-A71-infected tonsils.

## Discussion

EV-A71 is transmitted via fecal-oral, oral-oral, and respiratory routes but the primary site for EV-A71 replication and entry into human bloodstream remains poorly understood. The human palatine tonsils represent a mucosa-associated lymphoid tissue located in the oropharynx with a critical role in mucosal protection against alimentary and airborne pathogens including EV-A71. It has been presumed that the primary viral replication occurs in the lymphoid tissues of the oropharyngeal cavity (tonsils) and small bowel (Payer’s patches), followed by further replication in the regional lymph nodes (deep cervical and mesenteric nodes) leading to viremia [1,31]. In human autopsy tissues and animal models, EV-A71 is showed to spread to and within the central nervous system by axonal transport. Identification of EV-A71 in the squamous epithelium lining the tonsillar crypts and demonstration of the human tonsillar epithelial cell lines to be susceptible to EV-A71 [11,12] is of significance in further elucidating viral pathogenesis in patients. These observations suggest that human tonsillar epithelial cells may be novel and primary targets for EV-A71 infection and replication *in vivo* and EV-A71 could enter the central nervous system by axonal transport through facial nerve fibers, which innervates tonsils. In this study, we provide evidence that EV-A71 infection triggered intrinsic apoptosis in both tonsillar epithelial cell lines and various antiviral and proinflammatory cytokines were induced, indicating that the response of the tonsillar epithelium could be critical in exacerbating systemic as well as oropharyngeal symptoms in EV-A71 patients. However, without direct evidence the theory remains speculative whether EV-A71 could be transmitted into CNS through facial nerve axons innervating tonsils.

The dermotrophic properties of EV-A71 have been well known in patients since epidemics of HFMD in Japan in 1973 [32], who commonly developed vesicular exanthemas on the buccal mucosa, tongue, gums and palate and papulovesicular exanthemas on the hands, feet, and buttocks. The squamous epithelium lining the tonsillar crypts is usually subject to pathological change including edema, hyperaemia, herpes, and superficial ulcer. The mechanism by which a virus, such as EV-A71, causes these lesions has not been thoroughly studied.

Previous studies have identified EBV-encoded small RNA-1 in tonsillar consecutive specimens from tonsillectomies, suggesting that EBV may cause at least a portion of tonsillar atypical interfollicular hyperplasia in children and adolescents, which facilitated distortion of tonsillar architecture and virus spread [33]. Porcine reproductive and respiratory syndrome virus (PRRSV) was reported to induce apoptosis of lymphocytes in tonsils in pigs due to apoptosis [34]. Infection of human tonsillar tissue *ex vivo* with HIV-1 was sufficient to trigger apoptosis in CD4^+^ T cells during HIV-1 infection [35]. However, few studies have been carried out with regard to the role of tonsillar crypt epithelial cells in the local and systemic pathogenesis of EV-A71 infection.

Our data in this study demonstrated that intrinsic, but not extrinsic, apoptosis was triggered and confirmed that antiviral and proinflammatory cytokines were induced in EV-A71-infected human tonsillar epithelial cells. We hypothesize that EV-A71 invasion to human tonsillar epithelial cells and host response may critically contribute to viral pathogenesis as a result in patients. This may be particularly important in severe cases for failure to contain the virus in localized lesions in tonsils with rich blood circulation and facial nerve fiber innervation that could lead a potential breakthrough of the virus into the bloodstream and possible neurological complications. In fact, viral traveling and disseminating from tonsils to the central nervous system always has an advantage over other tissues or organs including the digestive tract for spatial proximity through either blood, lymphatic fluid, or facial nerve fibers.

In certain viral infections, apoptosis serves as an active defense mechanism for hosts to restrict virus replication and spread, especially at the early stage of infection. On the other hand, viruses could induce apoptosis to benefit their assembly, release, and spread at a late stage [36,37]. HIV-1 Vpr facilitated apoptosis by targeting the mitochondrial permeability transition pore, which might benefit the depletion of CD4^+^ lymphocytes [38]. HBV X protein induced apoptosis through sustained activation of cyclin B1-CDK1 kinase, likely contributing to cell growth inhibition [39].

EV-A71 infection induced apoptosis in various cell types through different mechanisms [40–42]. EV-A71 3C^pro^ promoted apoptosis through cleavage of PinX1 and caspase activation [43,44]. EV-A71 2A^pro^ induced apoptosis via cleavage of eukaryotic initiation factor 4G (eIF4G) [45]. EV-A71 2B is localized to the mitochondria which could induce mitochondrion-associated cell death signals by recruiting and directly interacting with the proapoptotic protein Bax [46]. EV-A71 also activated calpain via Ca^2+^ flux, which played an essential role in eliciting an apoptosis-inducing factor (AIF)-mediated, caspase-independent apoptotic pathway [47]. Here, we demonstrated that EV-A71-induced apoptosis in human tonsillar epithelial cells which was caspase-9 dependent. This mechanism for the mitochondria-mediated intrinsic apoptosis, activated in human tonsillar epithelial cells, differs in the process from the EV-A71-infected human lymphocytes and intestinal epithelial cells infected as described above, but is comparable to that in infected human astrocytes.

As for the mechanism by which intrinsic apoptosis was induced, our results showed that anti-apoptotic Bcl-2 and survivin were downregulated but the pro-apoptotic Bax increased in both cell lines (Fig 4A–4D). On the other hand, FLIP appeared not to be involved in activating and suppressing the apoptosis (Fig 4). Interestingly, caspase-8 was not cleaved and the extrinsic apoptotic pathway was not activated in UT-SCC-60A and UT-SCC-60B cells infected with EV-A71. We did not detect a significant amount of Bcl2 in mitochondria, so that Bcl2 could be ruled out to play an inhibitory effect on the induced intrinsic apoptosis. Survivin appeared to be present in the tonsillar epithelial cell lines and may inhibit the apoptosis by interacting directly with pro- caspase-3 or caspase-7 for blocking their cleavage to a certain degree. This inhibition may be relieved, and the cell death increased when the level of survivin declined in the late stage of infection.

The present study showed that IFN-β and IL-6 responses were induced in human tonsillar epithelial cells. CCL5, as a member of chemokines, was induced only in UT-SCC-60A cells whereas TNF-α was induced in both cell lines, albeit at a low level. The observation that elevated levels of IL-6, IL-8, and IP-10 along with higher white blood cell counts in the plasma and cerebrospinal fluid from severe EV-A71 patients than mild patients and normal controls suggests that innate immunity to EV-A71 was functional in human tonsillar epithelial cells. Upregulation of proinflammatory cytokines may demonstrate a more robust host response locally to contain the infection in tonsillar epithelial cells, which causes tonsillitis clinically in patients. This may occur early during the infection. If the infection is localized and cured timely due to effective antiviral immunity, the tonsillitis could be restricted and the virus invasion into the facial nerve fibers that innervate tonsils could be restrained. In contrast, if the immunity is compromised, the pathogenicity in EV-A71-infected tonsils might be able to affect the overall disease course leading to complications in the central nerve system. On the other hand, the inflammatory mediators, such as IL-6 and CCL5, released by tonsillar epithelial cells, could enter into the circulatory system via capillary network in the crypts and have impact on the Blood Brain Barrier (BBB) which may activate monocytes/macrophages, dendritic cells, natural killer cells, and lymphocytes and exacerbate the complications in the CNS. However, we tend to believe that the cytokines produced in tonsils would have limited impact directly on the pathogenicity in the central nervous system, in which the outcome would mainly result from viral invasion and subsequent immune responses occurring in the brain.

We observed that more robust IFN-β, IL-6, and CCL5 responses induced in UT-SCC-60A cells than in UT-SCC-60B cells. The UT-SCC-60B cells appeared to be more resistant in their responses and the differences between the two cell lines may result from various reasons. Divergence in distribution density of receptors for EV-A71 (including PSGL-1, SCARB2) and/or expression efficacy of host genes necessary for virus replication and host defense or proinflammatory response between two cell lines could be some of the causes. Unlike UT-SCC-60A, UT-SCC-60B was a tonsil squamous cell line originated from the cell that was metastasized to a lymph node. Many genes involved in innate immunity might have undergone mutations during and after migration under distinct microenvironment, which in turn caused altered transcription, translation and secretion of the cytokines and chemokines.

Our results in this study support that the MAPK (ERK1/2, p38, JNK1/2) pathways were involved in the regulation of cytokines in EV-A71-infected human tonsillar epithelial cells. Interestingly, cytokine responses in UT-SCC-60A cells following EV-A71 infection was more intensive than in UT-SCC-60B cells and ERK1/2 might play a more important role in the innate antiviral immunity against EV-A71. Many viruses have been reported to induce MAPK/ERK upon infection. The activation of ERK1/2 has been observed during the infection period of a number of other virus including coxsackievirus B3 [48], HIV-1 [49], hepatitis C virus [50], herpes simplex virus type 2 and respiratory syncytial virus [51,52]. Extensive involvement of this pathway suggests the presence of a global host strategy to regulate its response to viral infections in an early stage of infection. Further studies are underway to elucidate the molecular mechanism about how apoptosis and cytokine induction are regulated in human tonsillar epithelial cells infected by EV-A71, which will no doubt enhance our understanding of viral pathogenesis in EV-A71 patients.

## Supporting information

S1 Table Sequences of the primers used in the study. (DOCX)

S1 File Raw images for western blot analyses performed in the study. (PDF)
