# Supplementary material for: Intrinsic apoptosis and cytokine induction regulated in human tonsillar epithelial cells infected with enterovirus A71
Source: PLoS One. 2021 Jan 22;16(1):e0245529. doi: 10.1371/journal.pone.0245529 (PMC7822318; doi:10.1371/journal.pone.0245529)
Supplement: S1 Table — (DOCX) [file pone.0245529.s001.docx]

**Supplemental Table 1 Sequences of the Primers Used in the Study**

| Primer Name |  | Sequences（5’­3’） |
| --- | --- | --- |
| FasL | Forward | ATTTAACAGGCAAGTCCAACTCA |
|  | Reverse | GGCCACCCTTCTTATACTTCACT |
| IFN-α | Forward | GCCTCGCCCTTTGCTTTACT |
|  | Reverse | CTGTGGGTCTCAGGGAGATCA |
| IFN-β | Forward | GCTTGGATTCCTACAAAGAAGCA |
|  | Reverse | ATAGATGGTCAATGCGGCGTC |
| IFN-λ1 | Forward | CACATTGGCAGGTTCAAATCTCT |
|  | Reverse | CCAGCGGACTCCTTTTTGG |
| ISG-54 | Forward | AAGCACCTCAAAGGGCAAAAC |
|  | Reverse | TCGGCCCATGTGATAGTAGAC |
| CCL5 | Forward | CCAGCAGTCGTCTTTGTCAC |
|  | Reverse | CTCTGGGTTGGCACACACTT |
| IL-18 | Forward | TCTTCATTGACCAAGGAAATCGG |
|  | Reverse | TCCGGGGTGCATTATCTCTAC |
| IP-10 | Forward | GTGGCATTCAAGGAGTACCTC |
|  | Reverse | TGATGGCCTTCGATTCTGGATT |
| TNF-α | Forward | CCTCTCTCTAATCAGCCCTCTG |
|  | Reverse | GAGGACCTGGGAGTAGATGAG |
| Bax | Forward | CCCGAGAGGTCTTTTTCCGAG |
|  | Reverse | CCAGCCCATGATGGTTCTGAT |
| Bid | Forward | ATGGACCGTAGCATCCCTCC |
|  | Reverse | GTAGGTGCGTAGGTTCTGGT |
| Survivin | Forward | AGGACCACCGCATCTCTACAT |
|  | Reverse | AAGTCTGGCTCGTTCTCAGTG |
| FLIP | Forward | TCAAGGAGCAGGGACAAGTTA |
|  | Reverse | GACAATGGGCATAGGGTGTTATC |
| GAPDH | Forward | CTGGGCTACACTGAGCACC |
|  | Reverse | AAGTGGTCGTTGAGGGCAATG |
