# Supplementary material for: Intrinsic apoptosis and cytokine induction regulated in human tonsillar epithelial cells infected with enterovirus A71
Source: PLoS One. 2021 Jan 22;16(1):e0245529. doi: 10.1371/journal.pone.0245529 (PMC7822318; doi:10.1371/journal.pone.0245529)
Supplement: S1 File — (PDF) [file pone.0245529.s002.pdf]

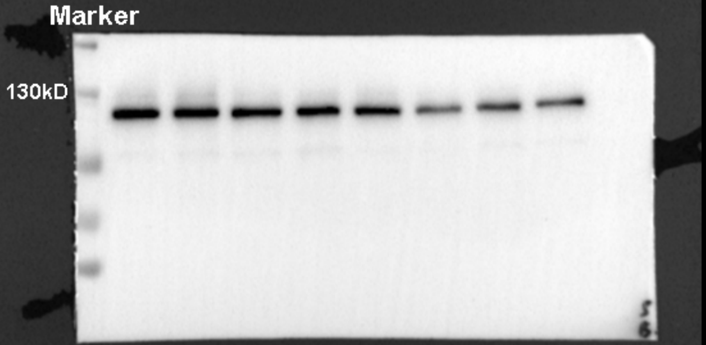

Fig.2A pro PARP

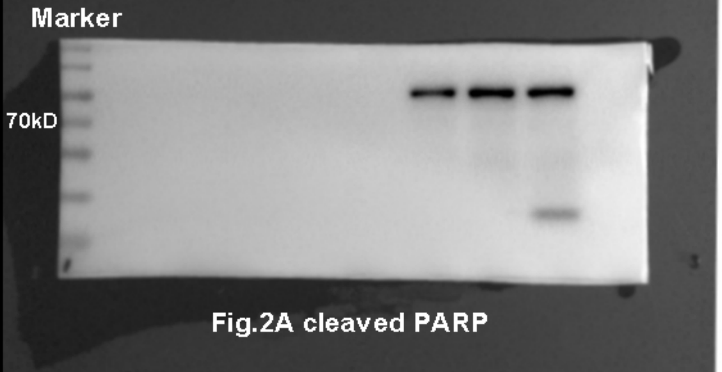

Fig.2A cleaved PARP

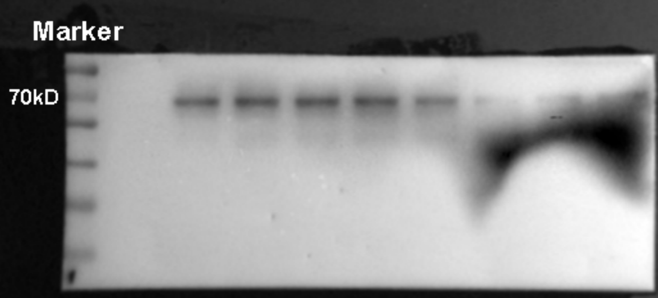

Fig. 2A pro Lamin A

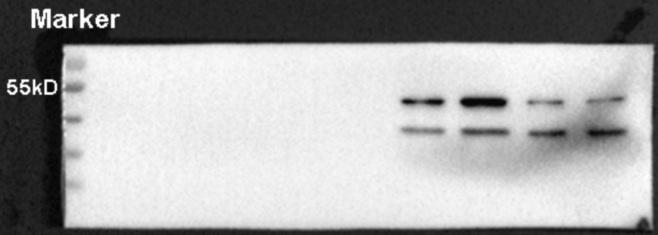

Fig.2A cleaved Lamin A

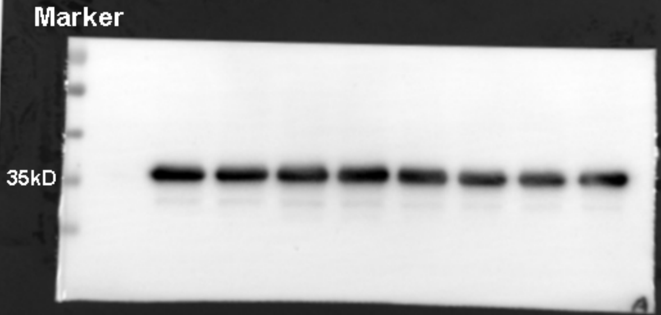

Fig. 2A pro caspase-7

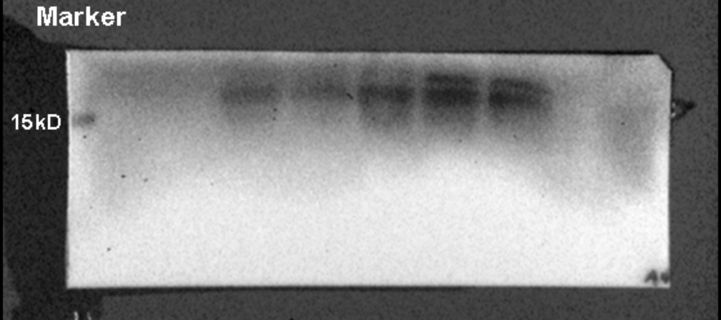

Fig.2A cleaved caspase-7

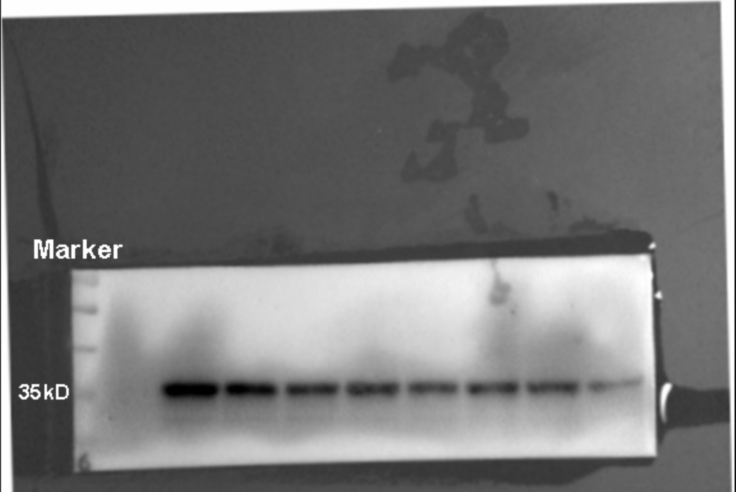

Fig.2A pro caspase-6

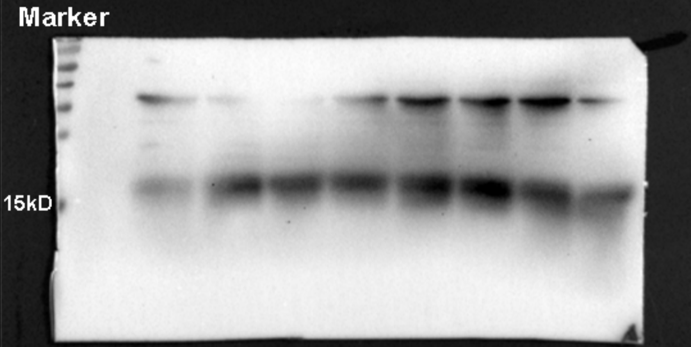

Fig.2A cleaved caspase-6

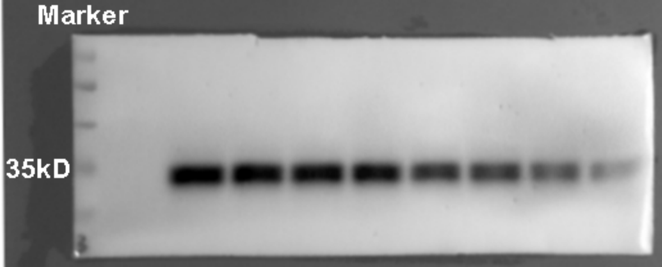

Fig.2A pro caspase-3

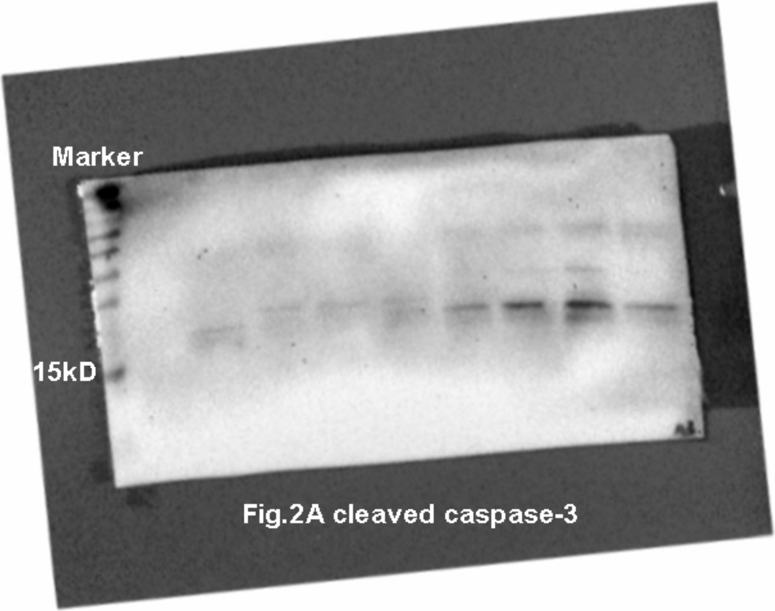

Fig.2A cleaved caspase-3

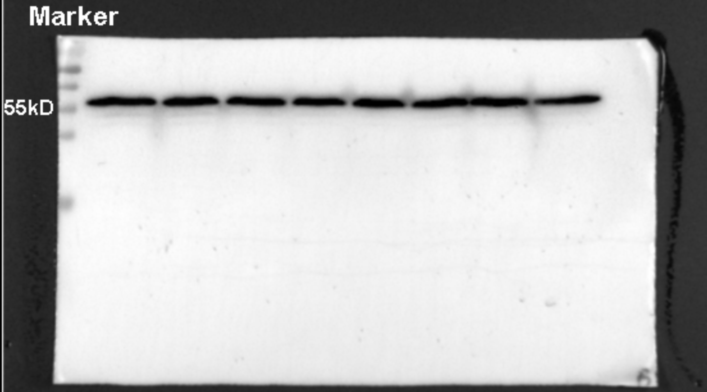

Fig.2A β-tubulin

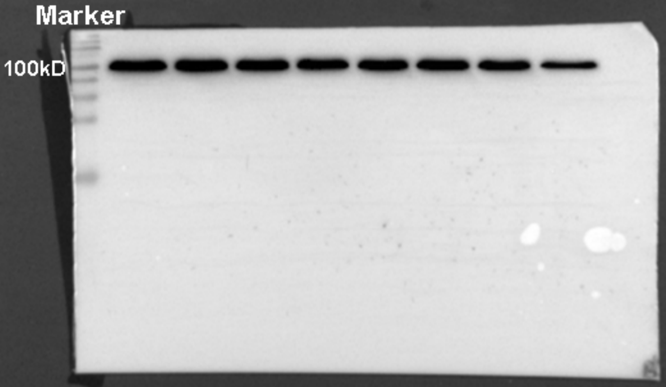

Fig. 2B pro PARP

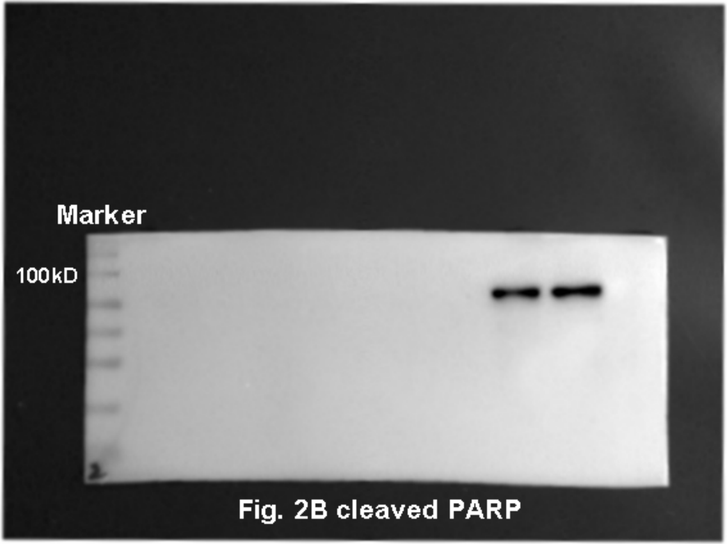

Fig. 2B cleaved PARP

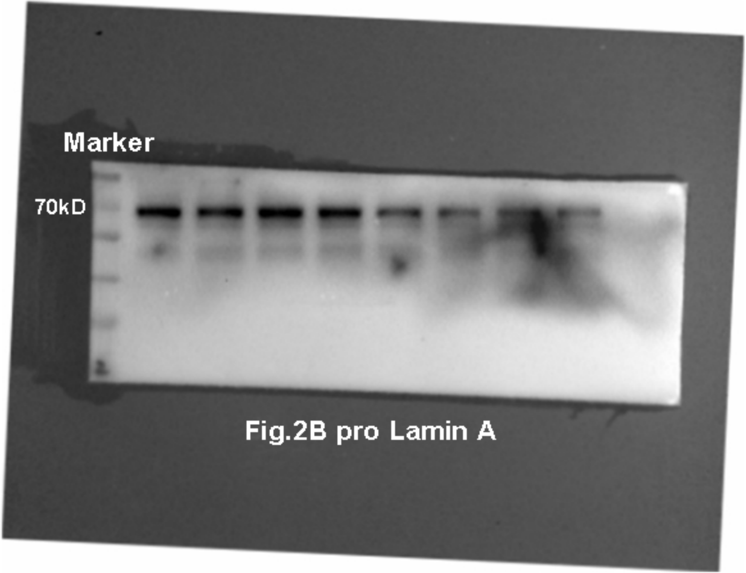

Fig.2B pro Lamin A

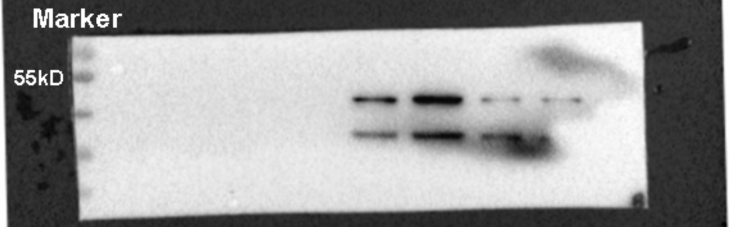

Fig. 2B cleaved Lamin A

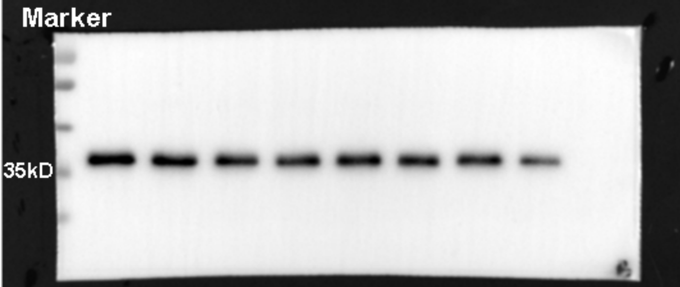

Fig. 2B pro caspase-7

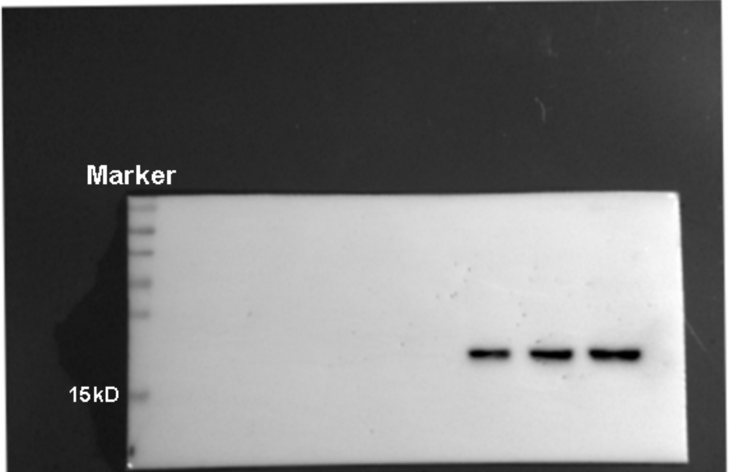

Fig. 2B cleaved caspase-7

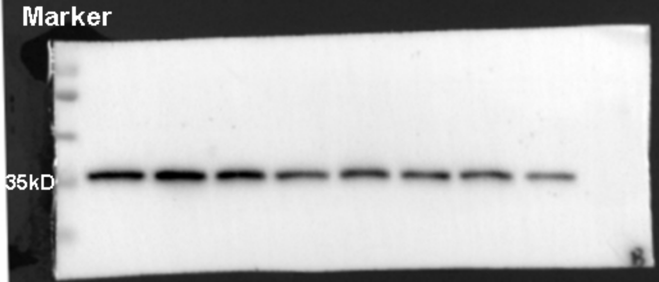

Fig. 2B pro caspase-6

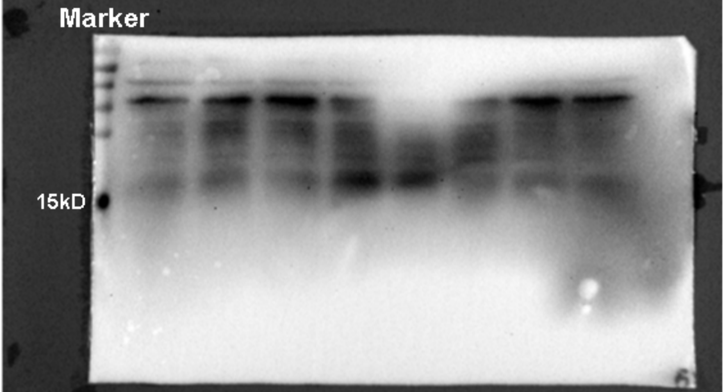

Fig. 2B cleaved caspase-6

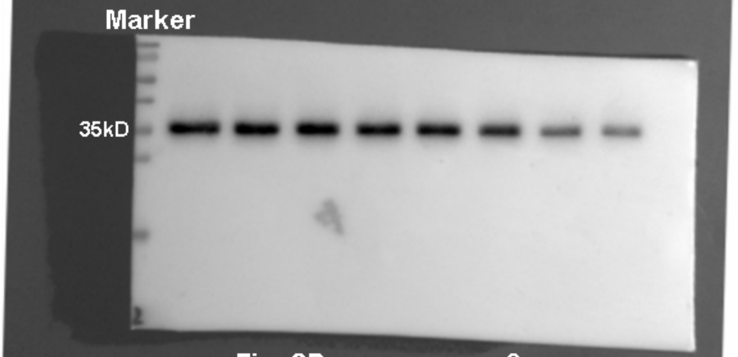

Fig. 2B pro caspase-3

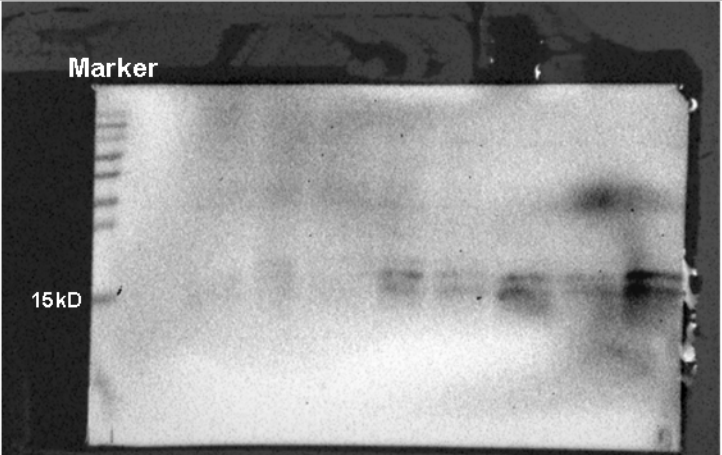

Fig.2B cleaved caspase-3

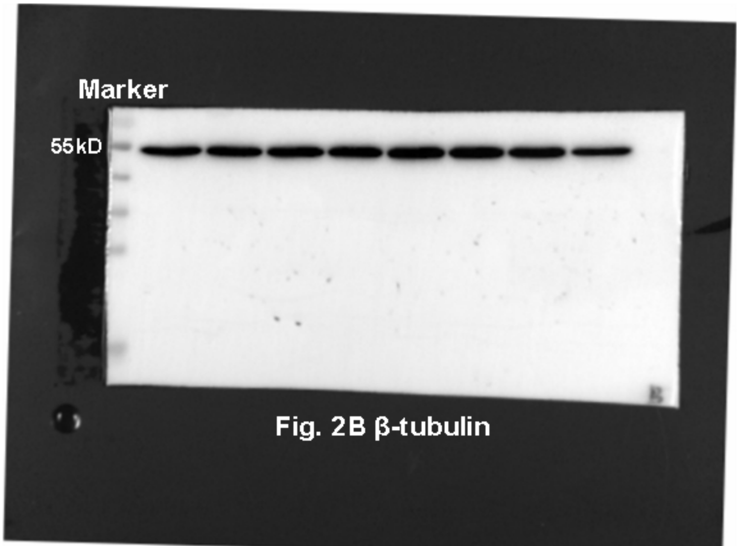

Fig. 2B β-tubulin

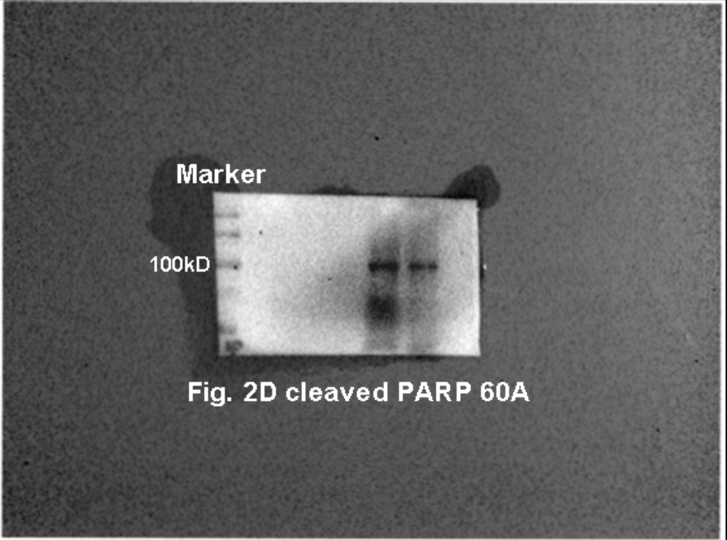

Fig. 2D cleaved PARP 60A

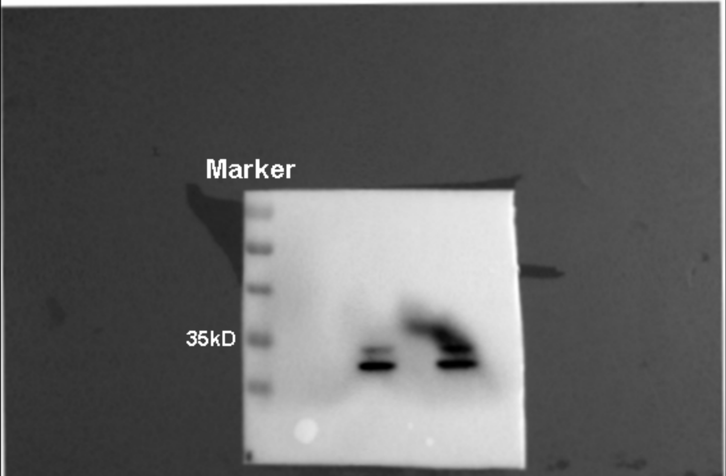

Fig. 2D cleaved Lamin A 60A

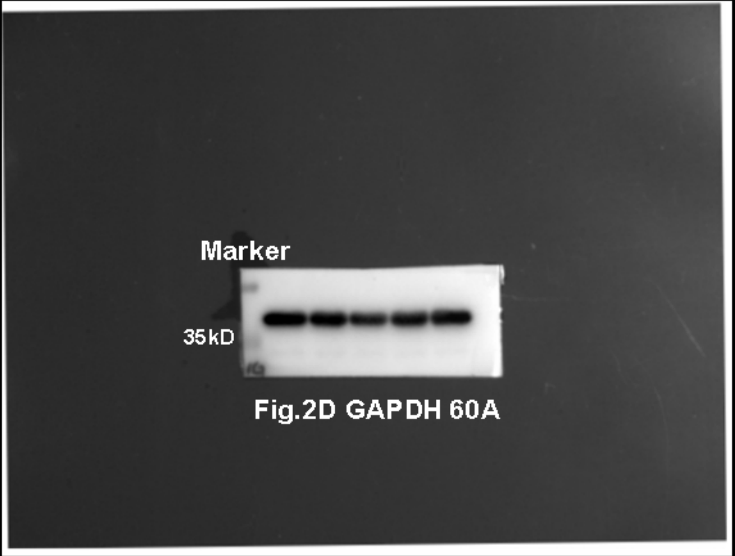
Marker
35kD
Fig.2D GAPDH 60A

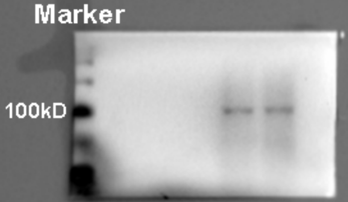

Fig. 2D cleaved PARP 60B

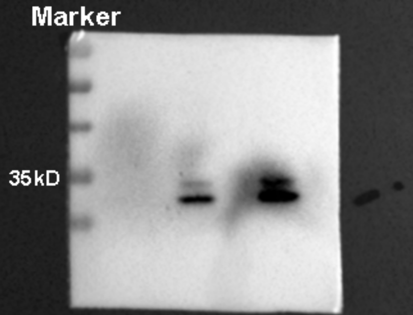

Fig. 2D cleaved Lamin A 60B

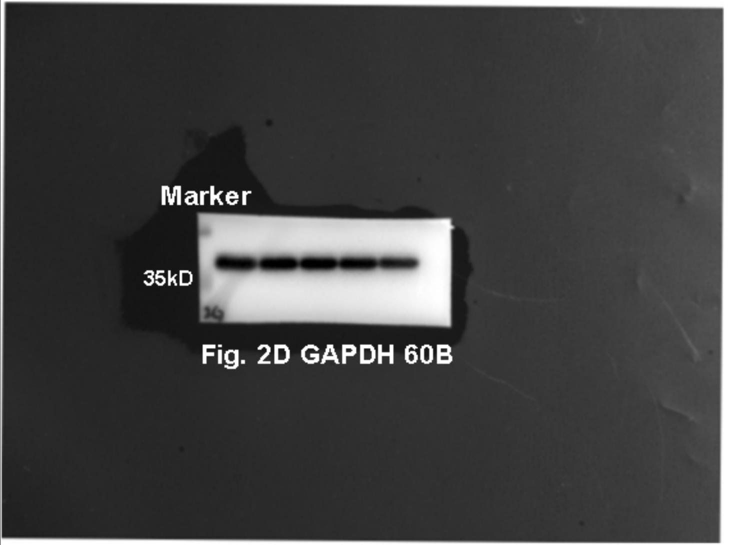

Fig. 2D GAPDH 60B

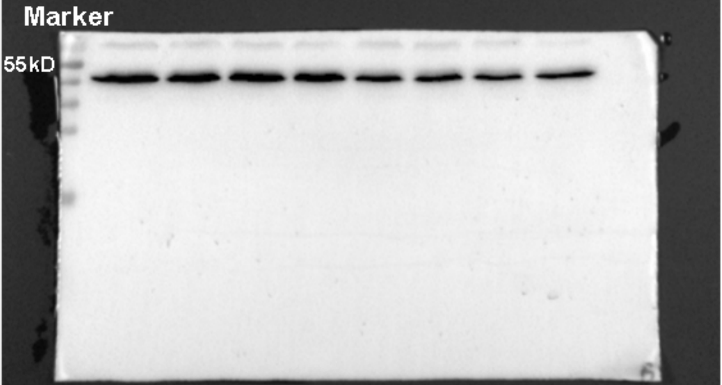

Fig.3A pro caspase-9

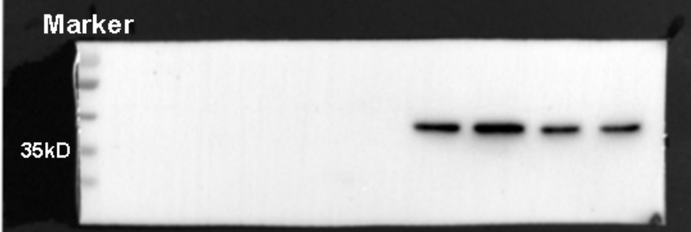

Fig. 3A cleaved caspase-9

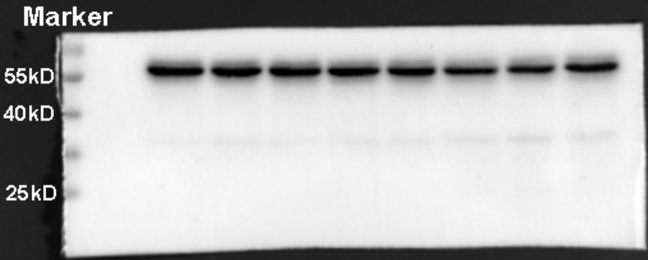

Fig. 3A pro and cleaved caspase-8

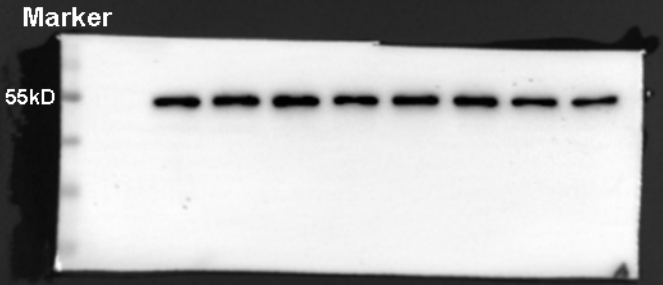

Fig. 3A pro caspase-2

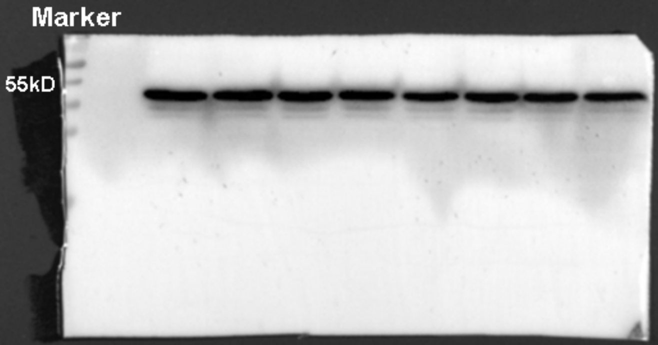

Fig. 3A β-tubulin

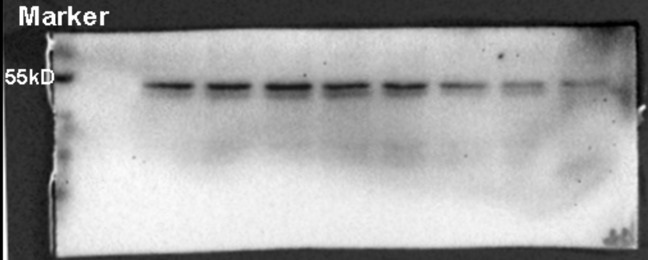

Fig. 3B pro caspase-9

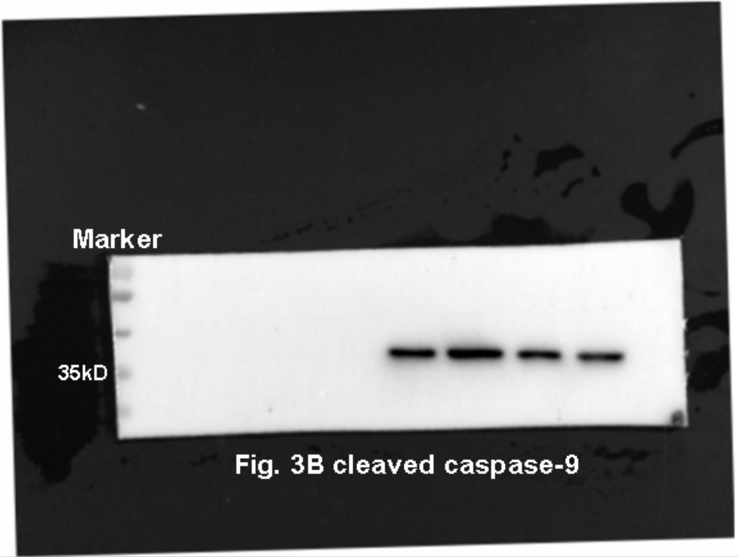
Marker
35kD
Fig. 3B cleaved caspase-9

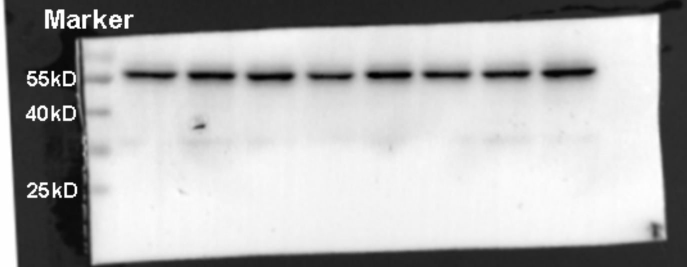

Fig. 3B pro and cleaved caspase-8

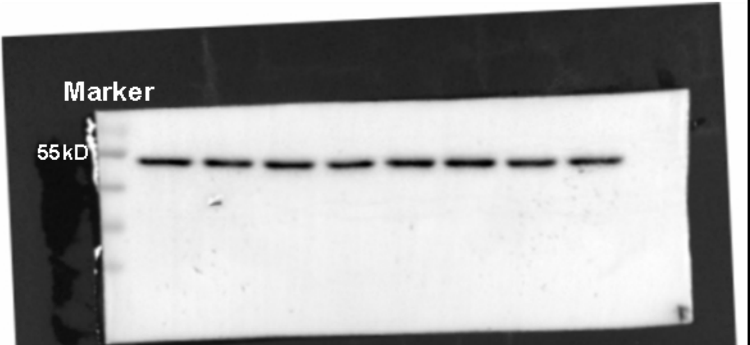

Fig. 3B pro caspase-2

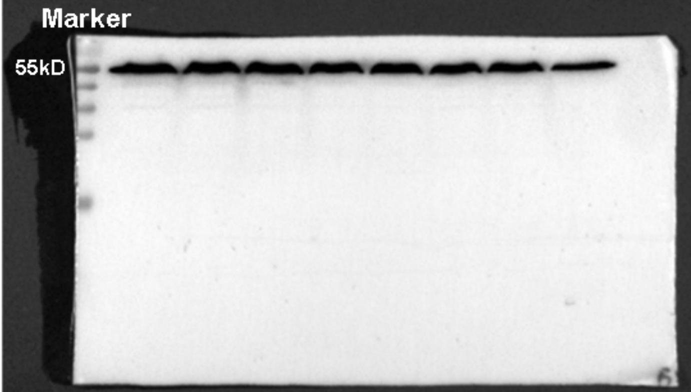

Fig.3B β-tubulin

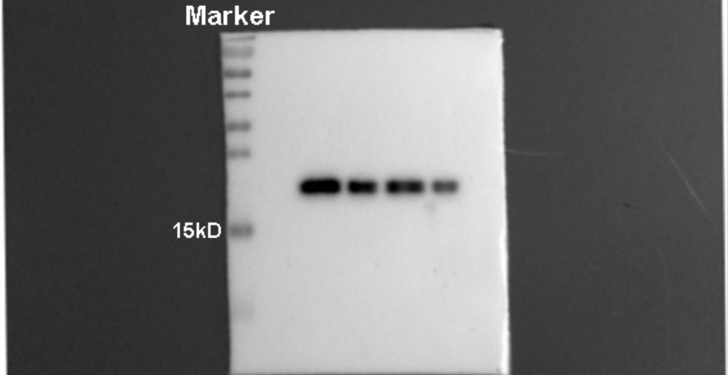

Fig.3E cleaved caspase-3 60A

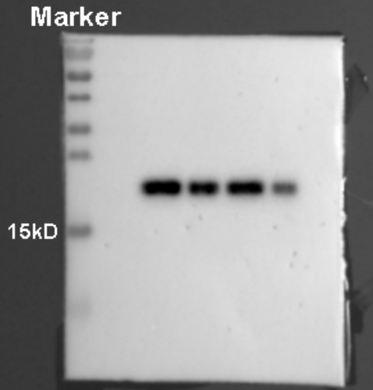

Fig. 3E cleaved caspase-6 60A

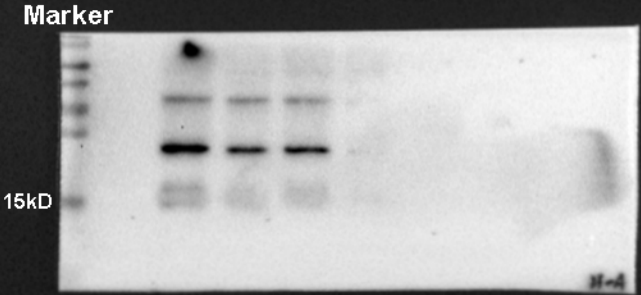

Fig.3E cleaved caspase-7 60A

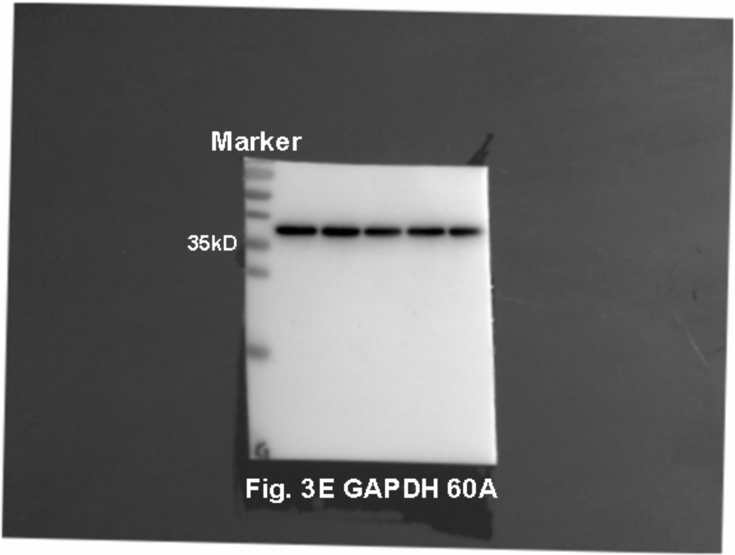

Fig. 3E GAPDH 60A

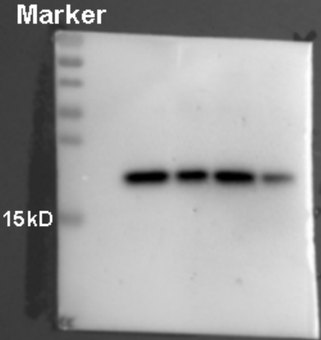

Fig. 3E cleaved caspase-3 60B

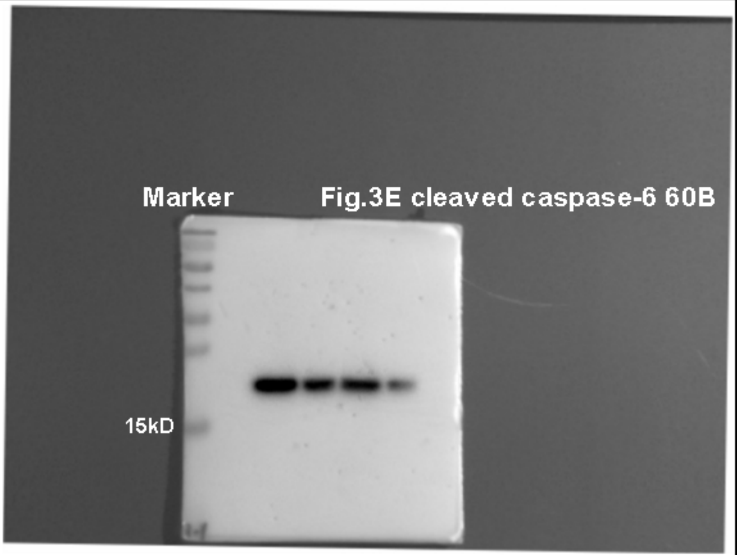
Marker
Fig.3E cleaved caspase-6 60B
15kD

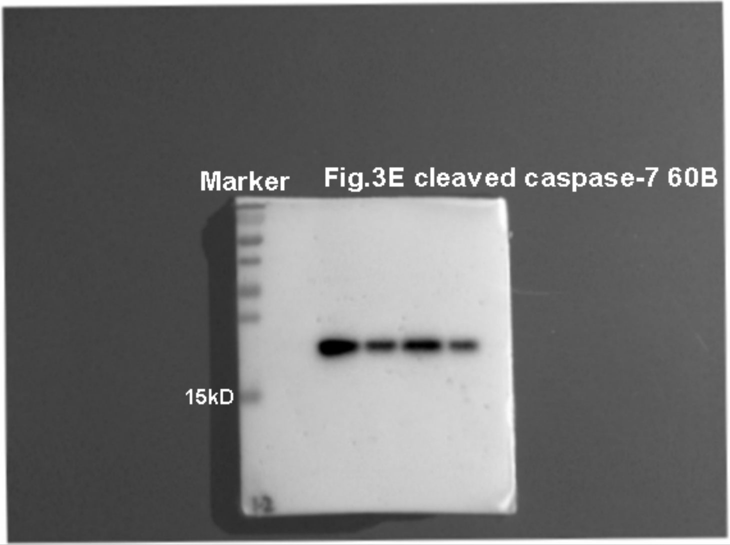
Marker
Fig.3E cleaved caspase-7 60B
15kD

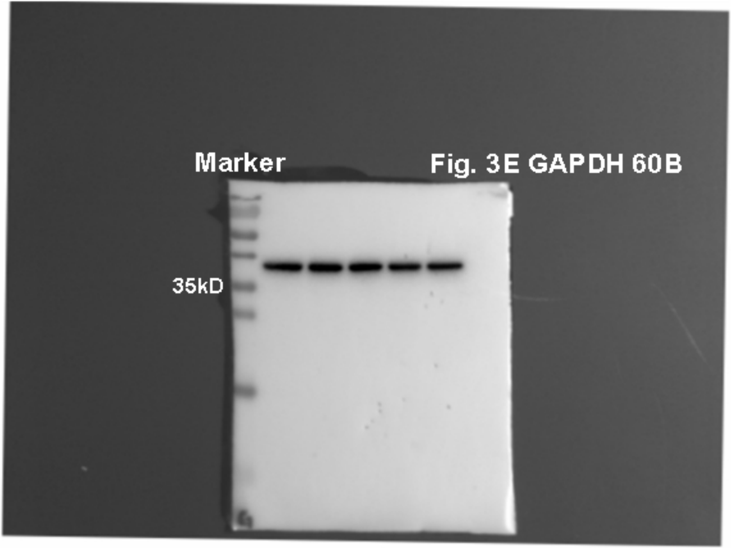
Marker
Fig. 3E GAPDH 60B
35kD

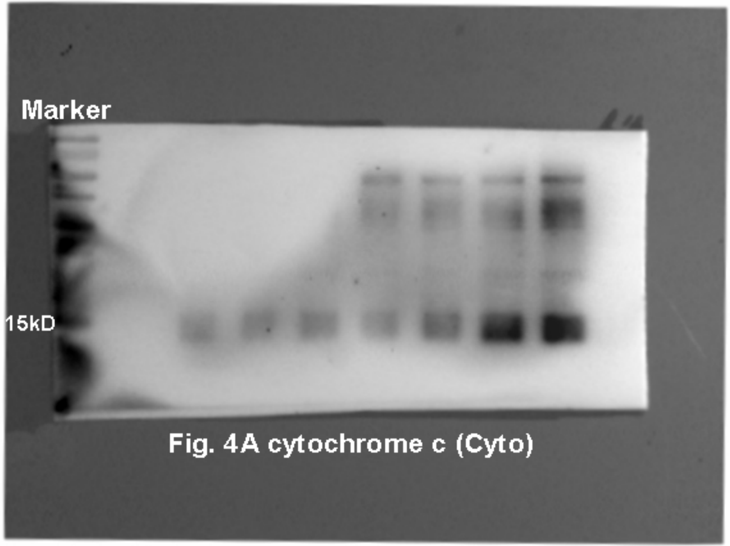

Fig. 4A cytochrome c (Cyto)

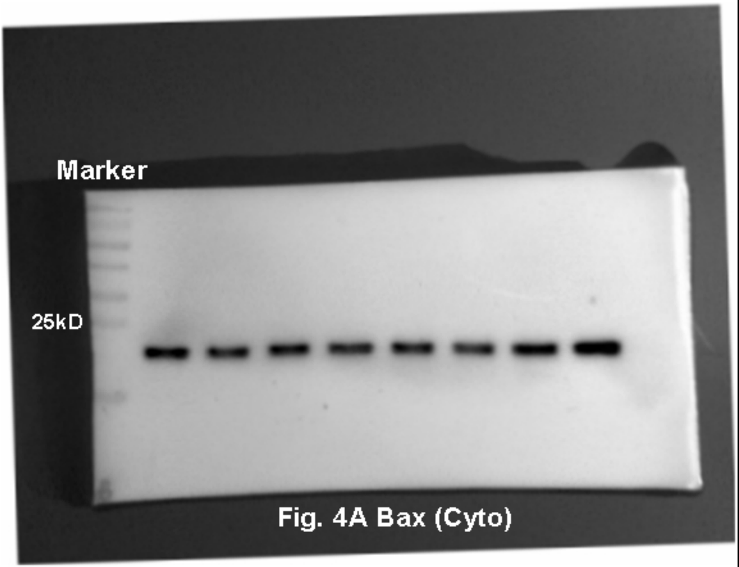

Fig. 4A Bax (Cyto)

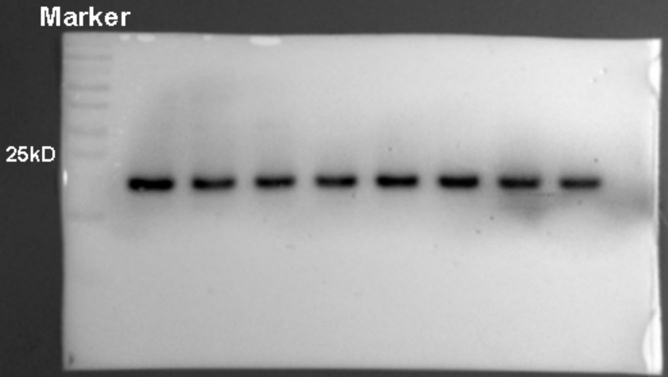

Fig. 4A Bid (Cyto)

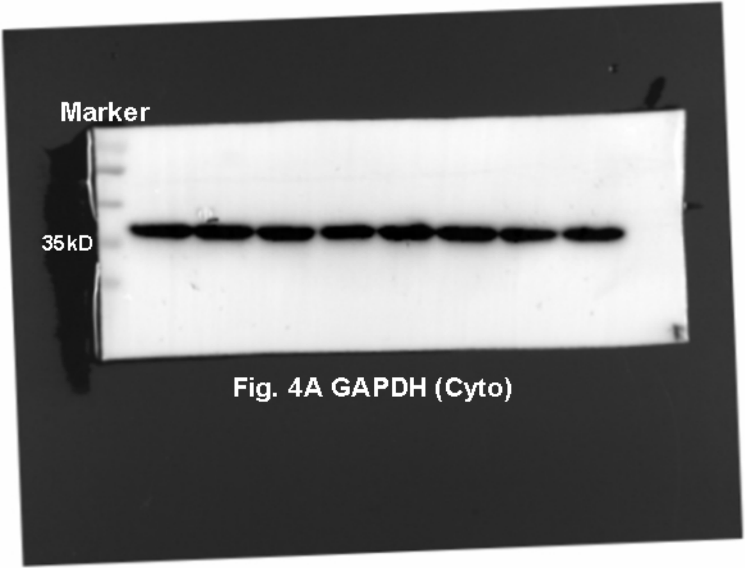

Fig. 4A GAPDH (Cyto)

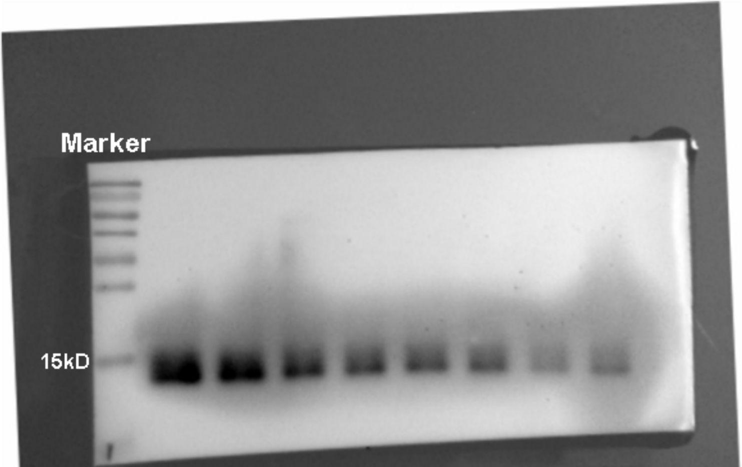

Fig. 4A cytochrome c (Mito)

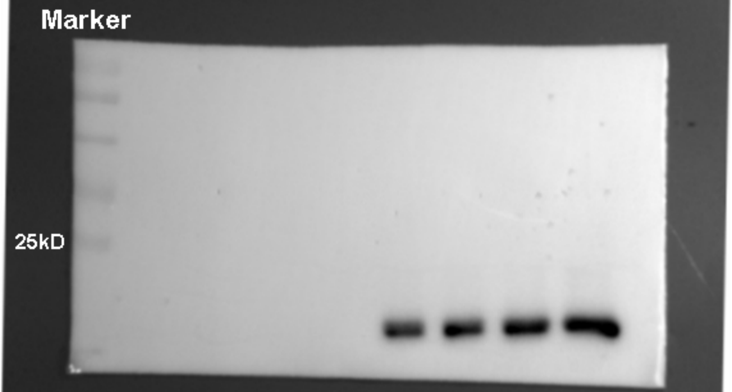

Fig. 4A Bax (Mito)

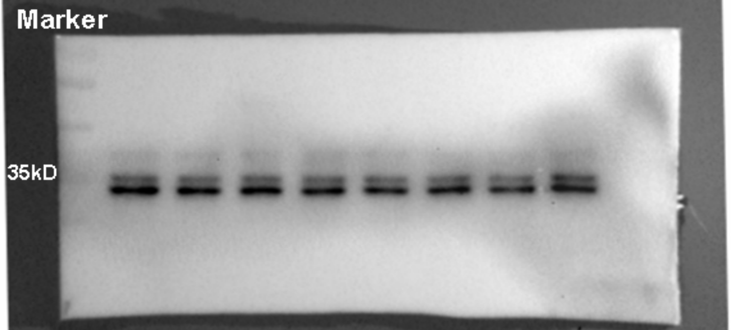

Fig. 4A VDAC1 (Mito)

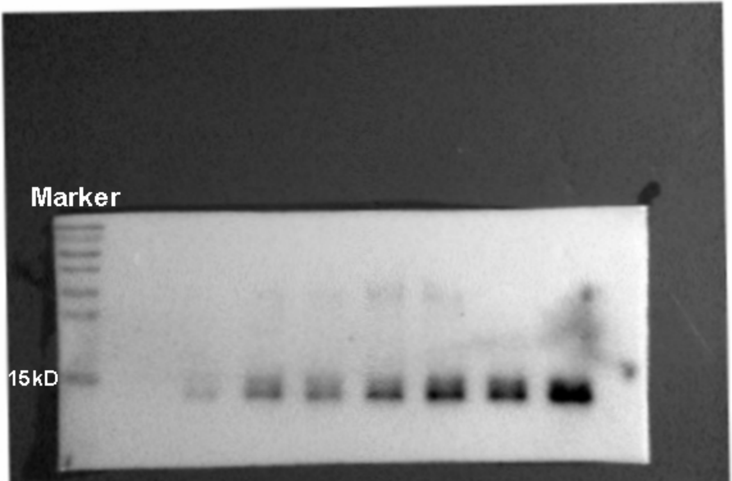

Fig. 4B cytochrome c (Cyto)

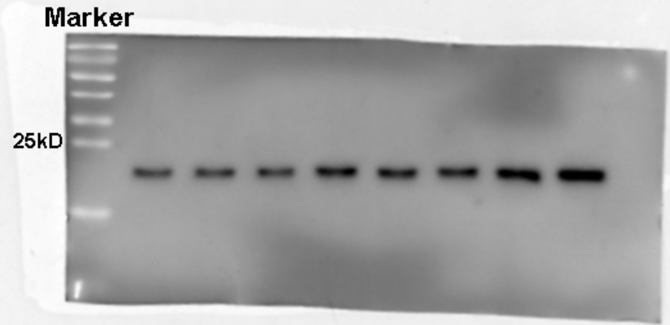

Fig. 4B Bax (Cyto)

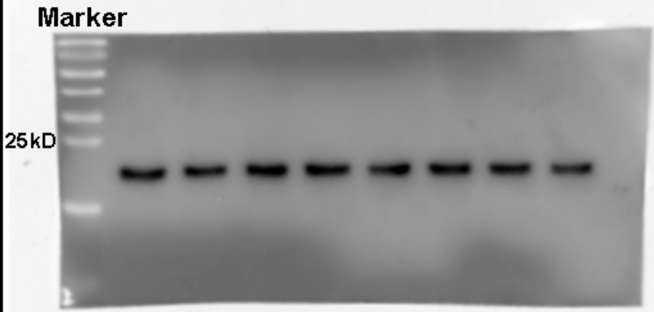

Fig. 4B Bid (Cyto)

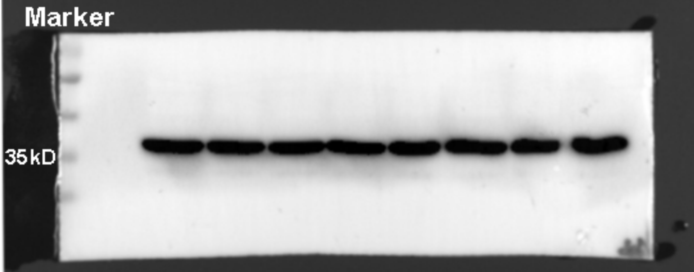

Fig. 4B GAPDH (Cyto)

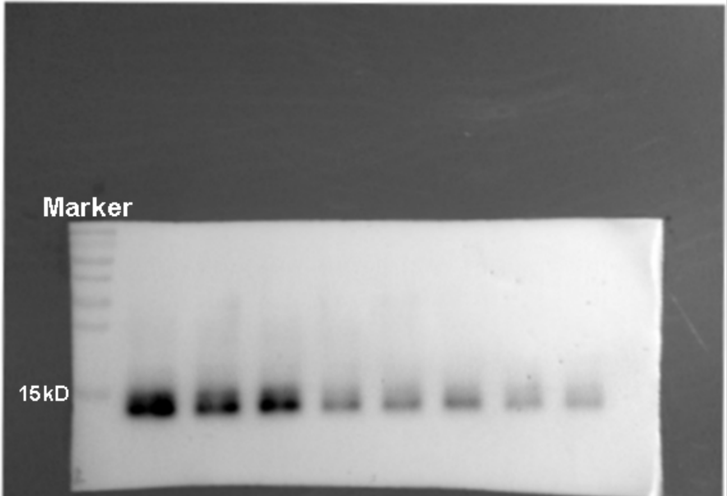

Fig. 4B cytochrome c (Mito)

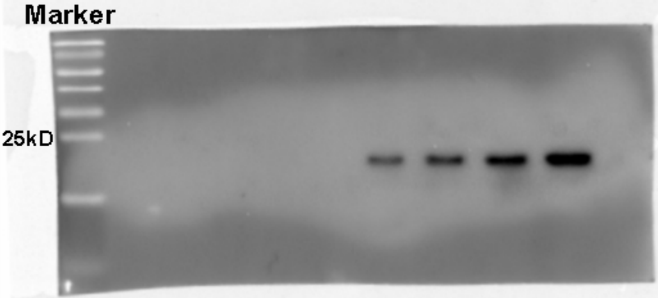

Fig. 4B Bax (Mito)

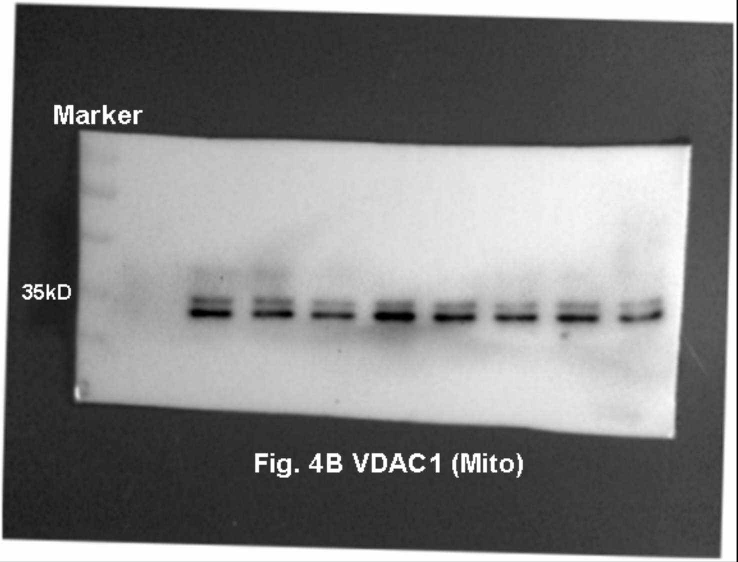

Fig. 4B VDAC1 (Mito)

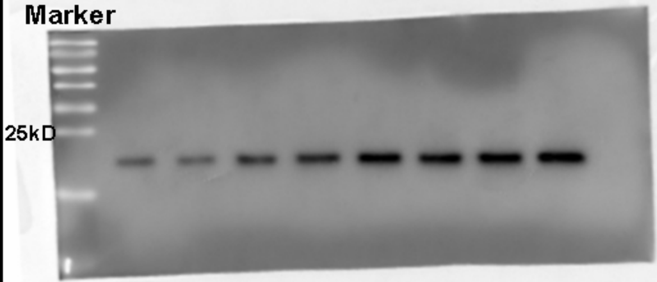

Fig. 4C Bax

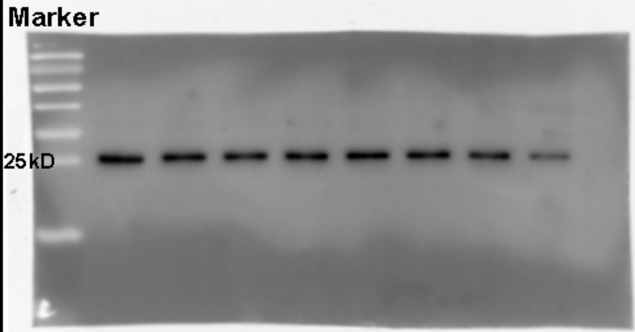

Fig. 4C Bcl-2

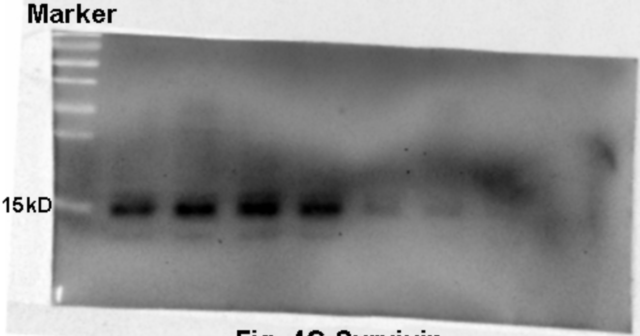

Fig. 4C Survivin

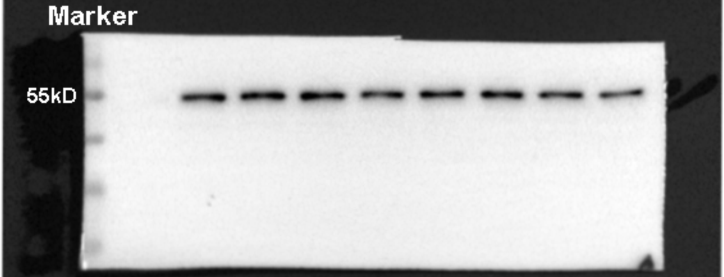

Fig. 4C FLIP

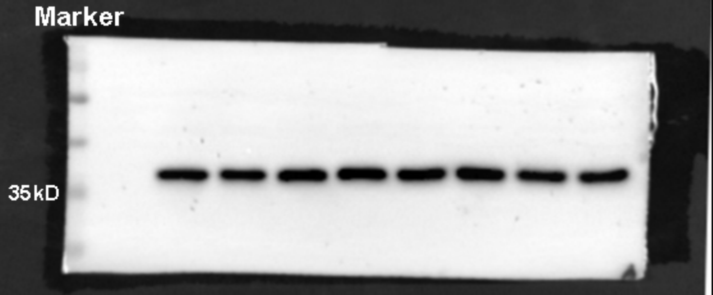

Fig. 4C GAPDH

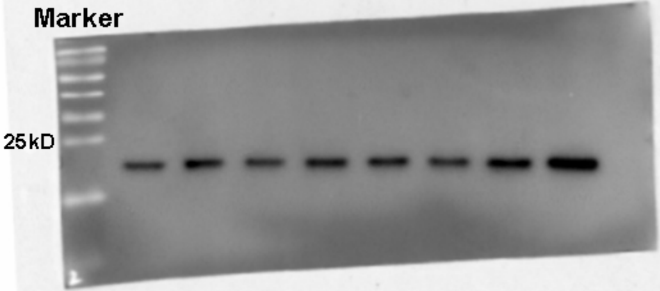

Fig. 4D Bax

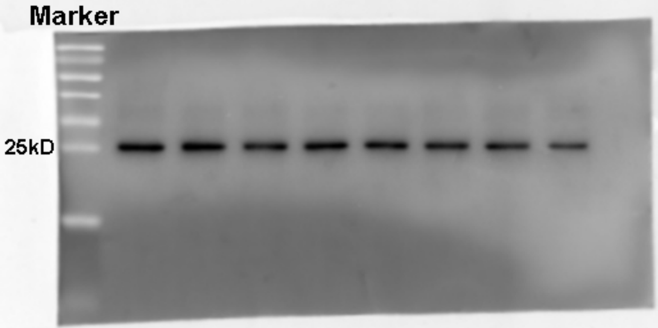

Fig. 4D Bcl-2

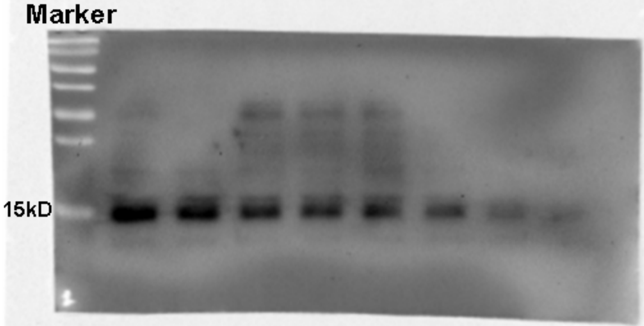

Fig. 4D Survivin

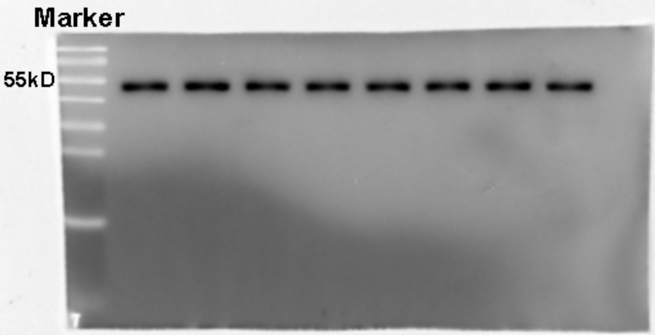

Fig. 4D FLIP

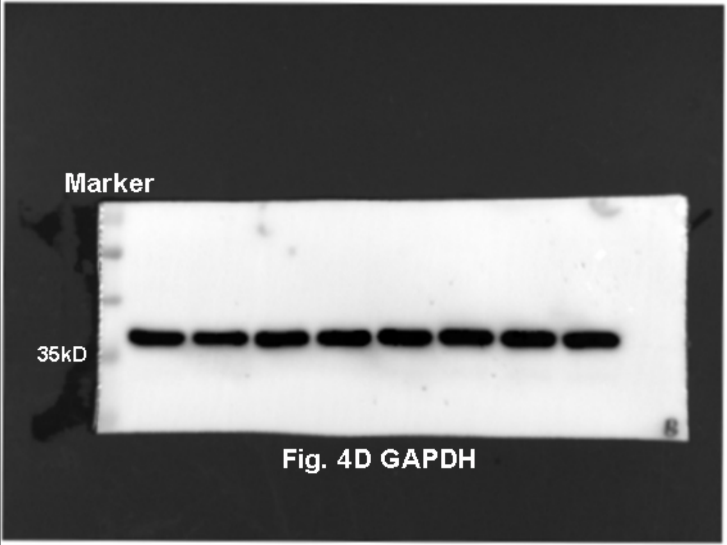

Fig. 4D GAPDH

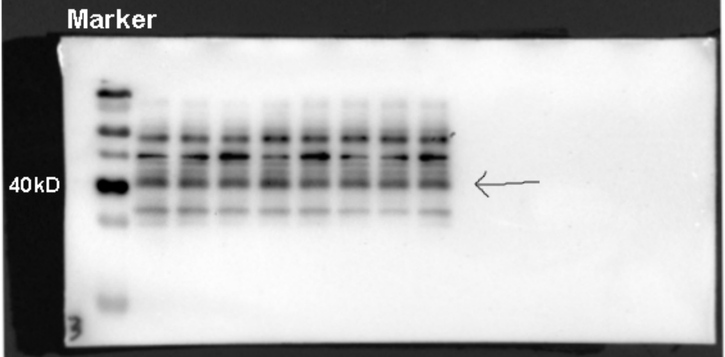

Fig. 5A pro caspase-1

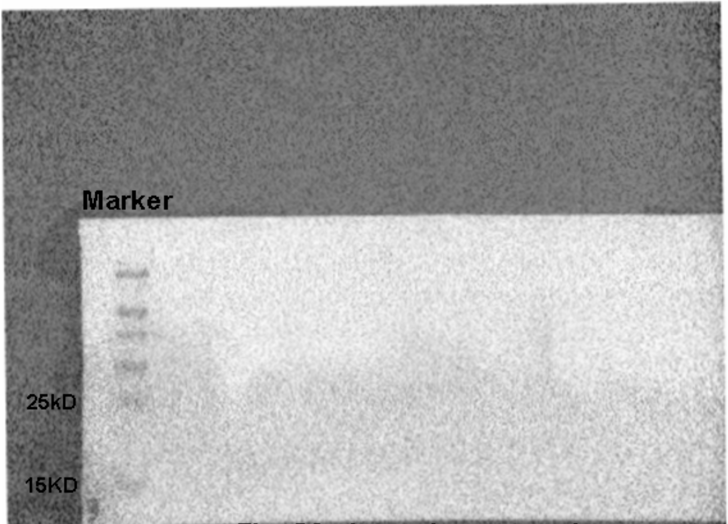

Fig. 5A cleaved caspase-1

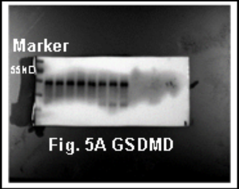

Fig. 5A GSDMD

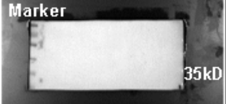

Fig. 5A cleaved GSDMD

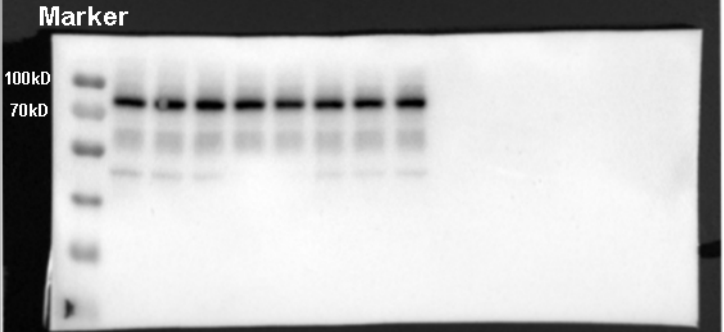

Fig. 5A RIPK1

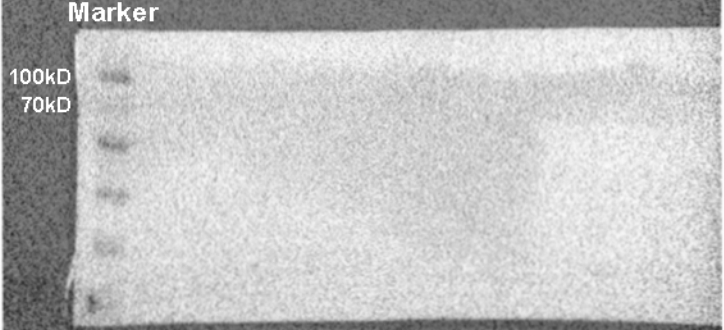

Fig. 5A pRIPK1

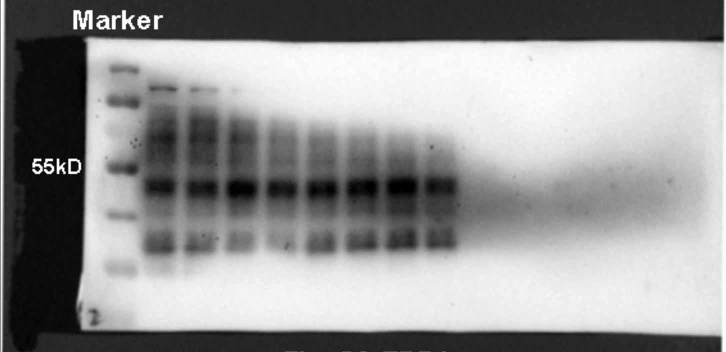

Fig. 5A ZBP1

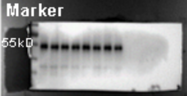

Fig. 5A MLKL

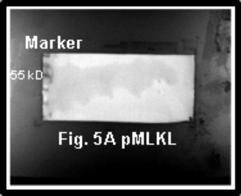
Marker
55 kD
Fig. 5A pMLKL

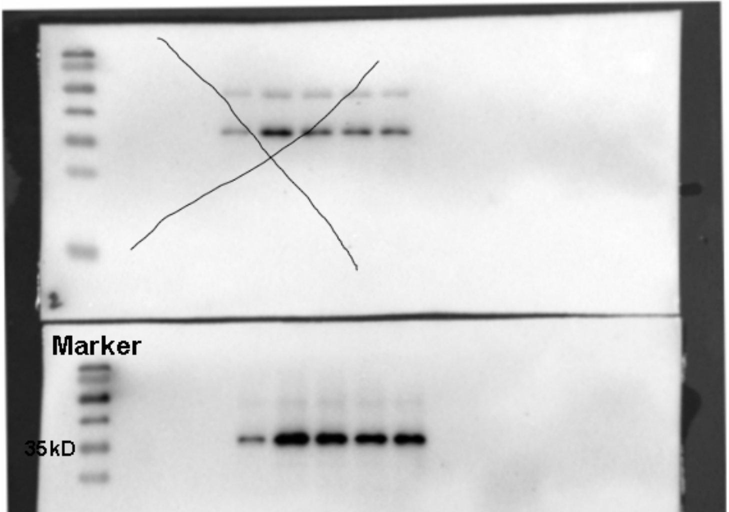

Fig. 5A VP1

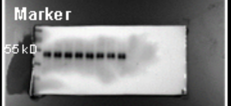

Fig. 5A β-tubulin

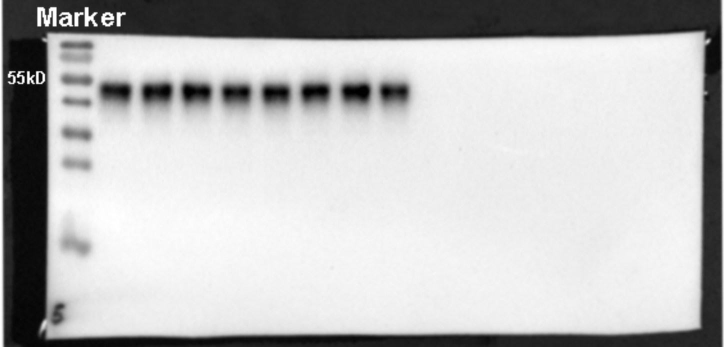

Fig. 5B pro caspase-1

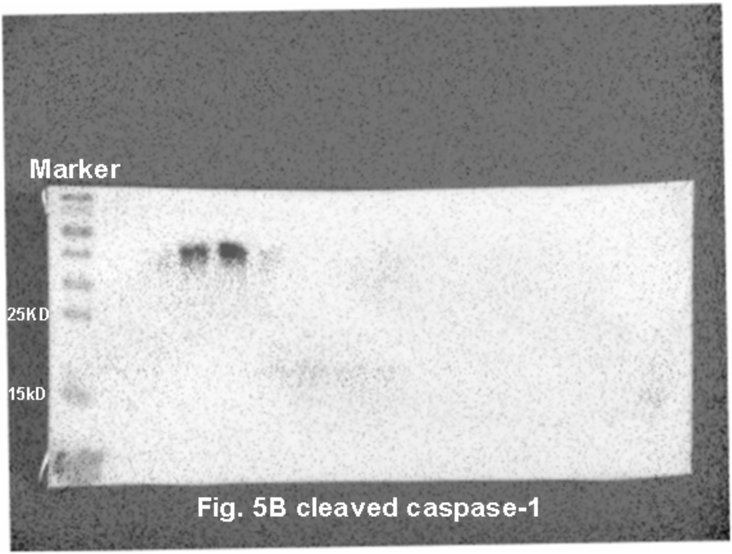

Fig. 5B cleaved caspase-1

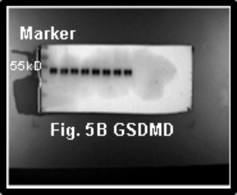
Marker
55kD
Fig. 5B GSDMD

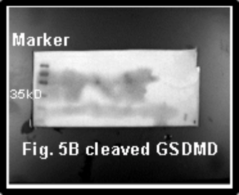

Fig. 5B cleaved GSDMD

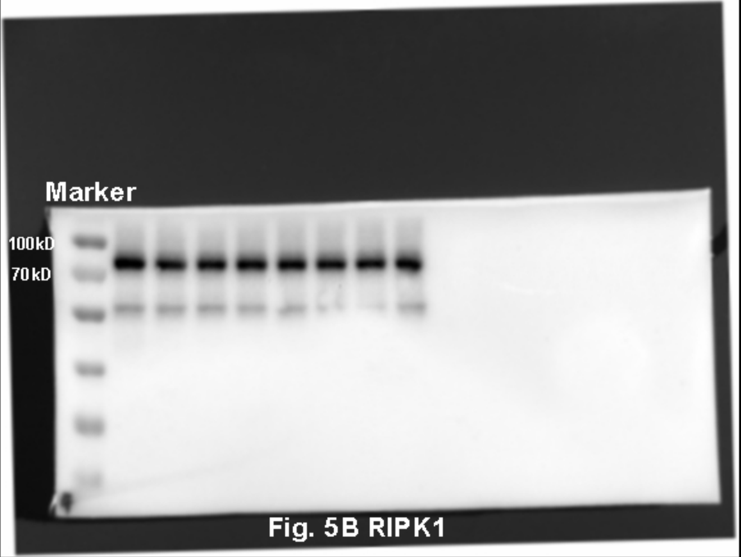

Fig. 5B RIPK1

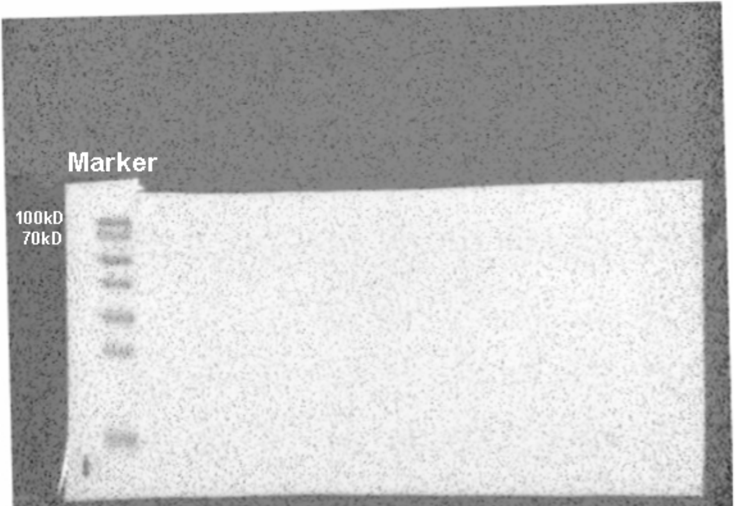

Fig. 5B pRIPK1

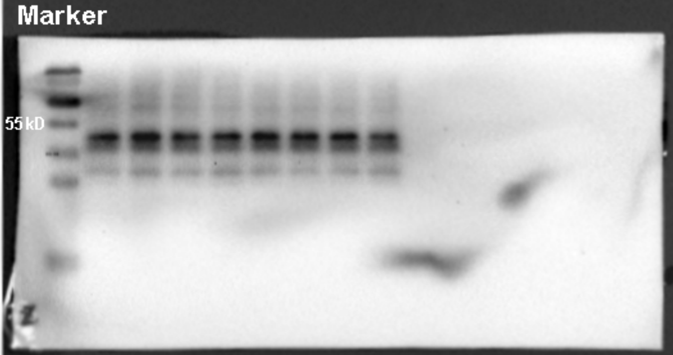

Fig. 5B ZBP1

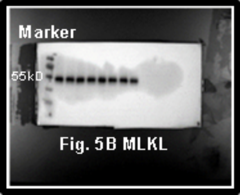
Marker
55kD
Fig. 5B MLKL

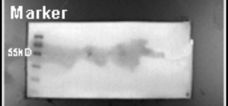

Fig. 5B pMLKL

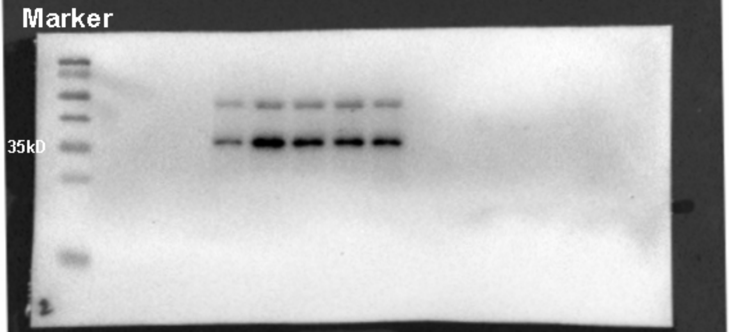

Fig. 5B VP1

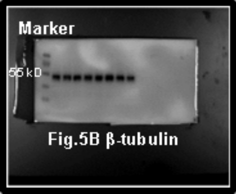

Fig.5B β-tubulin
